# Population genomics and morphometric assignment of western honey bees (*Apis mellifera* L.) in the Republic of South Africa

**DOI:** 10.1186/s12864-018-4998-x

**Published:** 2018-08-15

**Authors:** Amin Eimanifar, Samantha A. Brooks, Tomas Bustamante, James D. Ellis

**Affiliations:** 1 Honey Bee Research and Extension Laboratory, Entomology and Nematology Department, University of Florida, Gainesville, Florida, 32611-0620 USA; 2 Department of Animal Sciences, University of Florida, Gainesville, FL 32611 USA

**Keywords:** *Apis mellifera capensis*, *Apis mellifera scutellata*, Genetic differentiation, Population structure, GBS-SNP, Wing geometry, Standard morphometrics

## Abstract

**Backgrounds:**

*Apis mellifera scutellata* and *A.m. capensis* (the Cape honey bee) are western honey bee subspecies indigenous to the Republic of South Africa (RSA). Both bees are important for biological and economic reasons. First, *A.m. scutellata* is the invasive “African honey bee” of the Americas and exhibits a number of traits that beekeepers consider undesirable. They swarm excessively, are prone to absconding (vacating the nest entirely), usurp other honey bee colonies, and exhibit heightened defensiveness. Second, Cape honey bees are socially parasitic bees; the workers can reproduce thelytokously. Both bees are indistinguishable visually. Therefore, we employed Genotyping-by-Sequencing (GBS), wing geometry and standard morphometric approaches to assess the genetic diversity and population structure of these bees to search for diagnostic markers that can be employed to distinguish between the two subspecies.

**Results:**

*Apis mellifera scutellata* possessed the highest mean number of polymorphic SNPs (among 2449 informative SNPs) with minor allele frequencies > 0.05 (Np = 88%). The RSA honey bees generated a high level of expected heterozygosity (*H*_exp_ = 0.24). The mean genetic differentiation (*F*_ST_; 6.5%) among the RSA honey bees revealed that approximately 93% of the genetic variation was accounted for within individuals of these subspecies. Two genetically distinct clusters (*K* = 2) corresponding to both subspecies were detected by Model-based Bayesian clustering and supported by Principal Coordinates Analysis (PCoA) inferences. Selected highly divergent loci (*n* = 83) further reinforced a distinctive clustering of two subspecies across geographical origins, accounting for approximately 83% of the total variation in the PCoA plot. The significant correlation of allele frequencies at divergent loci with environmental variables suggested that these populations are adapted to local conditions. Only 17 of 48 wing geometry and standard morphometric parameters were useful for clustering *A.m. capensis*, *A.m. scutellata*, and hybrid individuals.

**Conclusions:**

We produced a minimal set of 83 SNP loci and 17 wing geometry and standard morphometric parameters useful for identifying the two RSA honey bee subspecies by genotype and phenotype. We found that genes involved in neurology/behavior and development/growth are the most prominent heritable traits evolved in the functional evolution of honey bee populations in RSA. These findings provide a starting point for understanding the functional basis of morphological differentiations and ecological adaptations of the two honey bee subspecies in RSA.

**Electronic supplementary material:**

The online version of this article (10.1186/s12864-018-4998-x) contains supplementary material, which is available to authorized users.

## Background

Within the insect family Apidae, western honey bees, *Apis mellifera* L. (Hymenoptera: Apidae), are cosmopolitan eusocial insects that play an important role in the cultivation of various crops and maintenance of healthy ecosystems globally [1, 2]. The genus *Apis*, which is comprised of nine honey bee species, is believed to have an evolutionary origin in Asia [3]. From there, honey bees have adapted to a diverse range of ecological conditions globally, diverging into eight Asian species and a ninth species, the western honey bee, which is endemic to Europe, Africa and the Middle East [4, 5]. Six evolutionary groups composed of about 25–30 subspecies of *A. mellifera* have been identified: (A) African subspecies, (M) northern and western European subspecies, (C) North Mediterranean subspecies, (O and Z) Middle Eastern subspecies, and (Y) in Ethiopia [4, 6–8].

Africa, specifically, is home to at least 11 *A. mellifera* subspecies distributed across the continent with substantial geographical variability among the areas in which the lineages are endemic [9]. It has been suggested that selective adaptation of honey bees to the huge variance of biotopes where they occur is the primary mechanism driving subspecies differentiation in Africa [10]. Two subspecies of African honey bees, *A.m. scutellata* from the Savannah areas of central and southern Africa, and *A.m. capensis* from the southern part of the Western and Eastern Cape of the Republic of South Africa (RSA), are of particular interest due to the behavioral characteristics they present [11].

Outside of its endemic range, *A.m. scutellata* is referred to as the “African,” “Africanized”, or “killer” bee of the Americas where it is considered invasive. *A.m. scutellata* and its hybrid populations have spread throughout South America, Central America and the southern parts of North America [12–14]. *Apis mellifera scutellata* exhibits several behaviors that beekeepers consider undesirable, but that are biologically important to the bee. These include excessive swarming, absconding, aggressive usurpation and heightened defensiveness [9, 12, 15]. Additionally, this bee competes for limited resources against, and hybridizes with, European-derived honey bees. These traits negatively impact beekeepers, bee colonies, and general public opinion of the honey bees. Furthermore, the ecological impact of *A.m. scutellata* in the Americas has not been quantified but is likely significant.

*Apis mellifera capensis* is a facultative social parasite that can reproduce thelytokously (unfertilized eggs can develop into diploid females). These bees are characterized by a unique set of genetic, behavioral and physiological traits expressed by the worker bees [9, 16–19]. The workers can develop into pseudoqueens (female bees that are neither queens nor workers, but possess qualities of both [20, 21]. Furthermore, they may have high number of ovarioles [22], well-developed spermathecae [23], shorter latency periods [24], and the ability to produce queen-like pheromones if a colony loses their queen [25, 26]. These traits facilitate the socially parasitic nature of some *A.m. capensis* workers (i.e. worker females can invade non-*A.m. capensis* honey bee colonies and become the resident reproductive female) [27, 28]. This led to the “*capensis* calamity” in the RSA when *A.m. capensis* colonies were moved by beekeepers to areas where *A.m. scutellata* were indigenous. Once there, *A.m. capensis* workers drifted into *A.m. scutellata* colonies and became social parasites of these colonies, thus leading to widespread colony collapse and the recognition of the threat *A.m. capensis* pose to non-*capensis* colonies [17].

The behavioral and ecological diversity of *A.m. scutellata* and *A.m. capensis* makes them ideal model organisms to investigate the genetic variability and population structure of African honey bee subspecies. Wild honey bees in sub-Saharan Africa are believed to have low levels of genetic differentiation which may be due to a high degree of panmixia and large dispersal capacity of colonies [11, 29]. The RSA subspecies are two exceptional cases, as they are reported to be structured genetically despite the lack of regional physical barriers existing between them [30]. Additionally, an intermediate zone between the distributions of *A.m. scutellata* and *A.m. capensis* is occupied by hybrids of the two subspecies [9]. Predictably, the hybrid bees have a mixed gene pool [17].

Knowledge concerning the population structure of *A.m. capensis* and *A.m. scutellata* and the stability of the hybrid zone over time is still incipient. Recent progress in the development of next-generation sequencing (NGS) platforms has enabled scientists to genotype large groups of individuals using a genotyping-by-sequencing (GBS) approach [31]. GBS is a highly multiplexed, high-throughput, low-cost method and is one of the simplest reduced representation genome approaches developed thus far [31, 32]. The large numbers of SNPs obtained with the GBS method result in an accurate assessment of genetic diversity and population structure and simplify the detection of adaptive putative loci associated with environmental pressure [33–35].

Herein, we investigated genetic differentiation and population structure within 464 *A.m. capensis*, *A.m. scutellata* and hybrid honey bees collected from 69 different apiaries, representing 28 geographical regions across the natural distribution of honey bees in the RSA. We also determined if allele frequencies at divergent loci were significantly correlated with environmental variables, in an effort to identify regions of the genome under natural selection. We further measured wing geometry and standard morphometric parameters to evaluate the differentiation pattern between *A.m. capensis* and *A.m. scutellata*, given that morphometrics is the current tool utilized to separate the subspecies [36] and the possibility that wing geometry could offer a quicker identification method with comparable accuracy [37, 38]. The resulting GBS and morphometric data provide information critically needed for designing diagnostic markers to differentiate between *A.m. capensis* and *A.m. scutellata* bees.

## Methods

### Honey bee collections

**Fig. 1:**
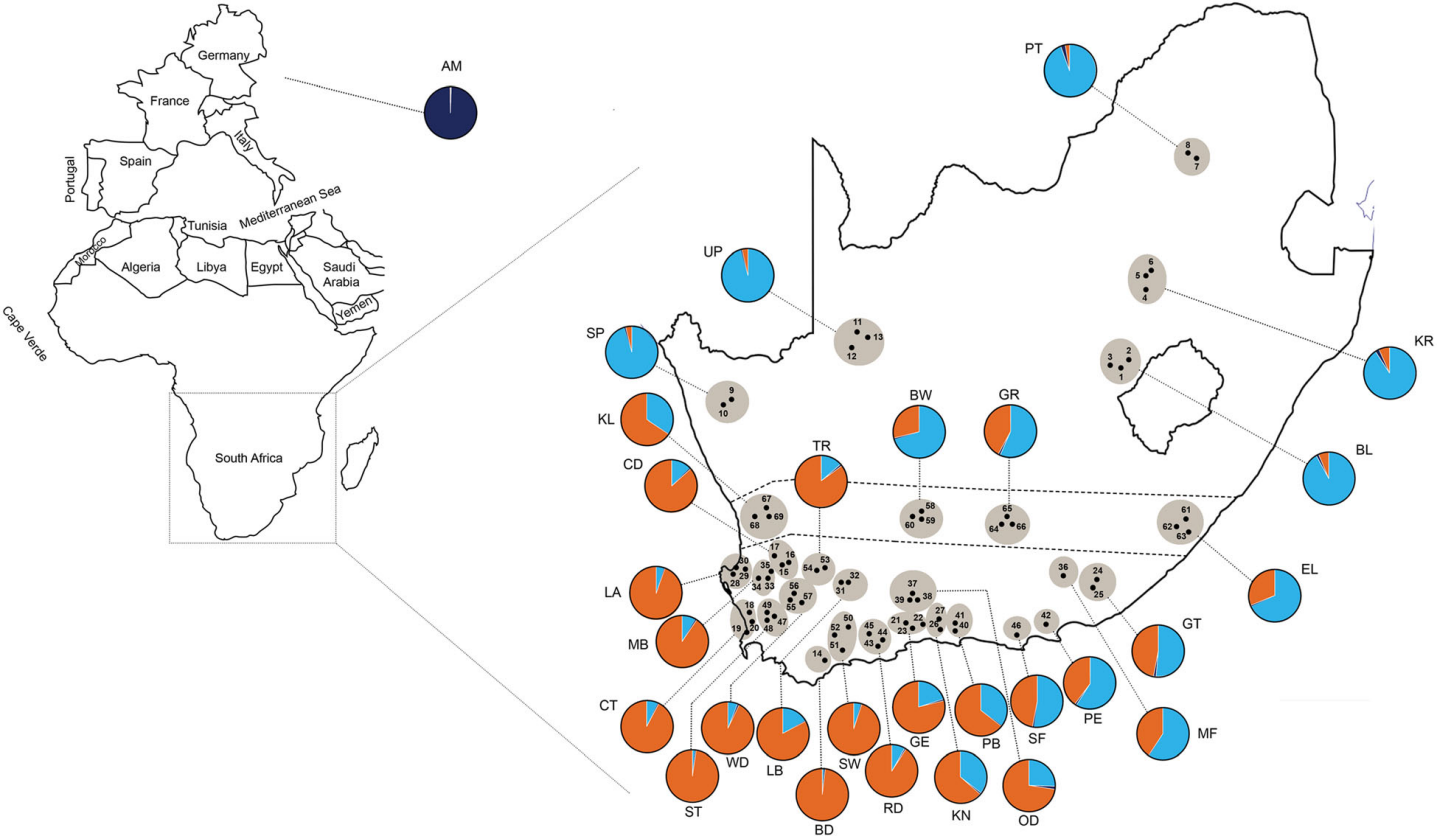
Geographic distribution of apiaries (*N* = 69) from which honey bees were collected in the Republic of South Africa. Adjacent apiaries are clustered into single geographical regions (*N* = 28) and assigned an abbreviation corresponding to the nearest town (corresponds to Table 1). The pie charts represent the composition of the three genetic clusters from each geographical region (shown as orange, blue, and dark blue). The colors indicate the different proportion of allele frequencies assigned to each region

We collected samples of worker honey bees from 1000+ managed colonies across the RSA in 2013 and 2014. These collections spanned the native geographical distribution of *A.m. capensis*, *A.m. scutellata* and hybrids of the two in the RSA. The number of bees examined per region/apiary and geographic information are reported in Table 1, Fig. 1. We analyzed between one and 21 bees per apiary, from 69 different apiaries, representing 28 geographical regions in and 464 bees from the RSA. Ten European-derived *A. mellifera* samples collected from honey bee colonies located at Honey Bee research Extension Laboratory, University of Florida were included in the genetic analysis for reference purposes. The RSA honey bees were collected into 50 ml vials containing absolute ethanol, imported into the US per USDA APHIS protocol and approval, and stored at − 20 °C prior to morphological and molecular analyses.

**Table 1:** Summary information for honey bee samples collected in the Republic of South Africa

No.	Geographic region - apiary	Apiary identifier	N	Geographical coordinates
1	Bloemfontein – A	BL – A	5	29.24°S – 26.95°E
2	Bloemfontein – B	BL – B	6	29.20°S – 27.20°E
3	Bloemfontein – C	BL – C	8	29.24°S – 26.94°E
4	Kroonstad – A	KR – A	6	27.58°S – 27.30°E
5	Kroonstad – B	KR – B	1	27.33°S – 27.50°E
6	Kroonstad – C	KR – C	10	27.27°S – 27.50°E
7	Pretoria – A	PT – A	3	25.74°S – 28.26°E
8	Pretoria – B	PT – B	6	25.70°S – 28.10°E
9	Springbok – B	SP – B	4	29.71°S – 17.78°E
10	Springbok – C	SP – C	5	29.67°S – 17.81°E
11	Upington – A	UP – A	5	28.48°S – 21.18°E
12	Upington – B	UP – B	5	28.72°S – 20.98°E
13	Upington – C	UP – C	9	28.52°S – 21.24°E
14	Bredasdorp – A	BD – A	6	34.50°S – 20.35°E
15	Citrusdaal – A	CD – A	8	32.86°S – 19.21°E
16	Citrusdaal – B	CD – B	8	32.84°S – 19.24°E
17	Citrusdaal – C	CD – C	11	32.67°S – 19.06°E
18	Cape Town – A	CT – A	5	33.80°S – 18.36°E
19	Cape Town – B	CT – B	3	33.97°S – 18.51°E
20	Cape Town – C	CT – C	12	33.96°S – 18.45°E
21	George – A	GE – A	9	33.90°S – 22.33°E
22	George – B	GE – B	8	33.95°S – 22.75°E
23	George – C	GE – C	8	33.98°S – 22.47°E
24	Grahamstown – A	GT – A	9	33.31°S – 26.49°E
25	Grahamstown – B	GT – B	6	33.37°S – 26.42°E
26	Knysna – A	KN – A	13	34.05°S – 22.99°E
27	Knysna – B	KN – B	19	34.02°S – 22.97°E
28	Langebaan – A	LA – A	10	33.04°S – 18.09°E
29	Langebaan – B	LA – B	6	33.00°S – 18.31°E
30	Langebaan – C	LA – C	6	33.03°S – 18.10°E
31	Laingsburg – A	LB – A	4	33.27°S – 20.85°E
32	Laingsburg – B	LB – B	6	33.28°S – 20.97°E
33	Moorreesburg – A	MB – A	2	33.10°S – 18.74°E
34	Moorreesburg – B	MB – B	3	33.11°S – 18.56°E
35	Moorreesburg – C	MB – C	2	33.02°S – 18.85°E
36	Modderfontein – A	MF – A	15	33.18°S – 25.80°E
37	Oudtshoorn – A	OD – A	1	33.50°S – 22.51°E
38	Oudtshoorn – B	OD – B	5	33.53°S – 22.54°E
39	Oudtshoorn – C	OD – C	2	33.58°S – 22.49°E
40	Plettenburg Bay – A	PB – A	3	34.05°S – 23.36°E
41	Plettenburg Bay – B	PB – B	12	34.09°S – 23.34°E
42	Port Elizabeth – A	PE – A	21	33.87°S – 25.39°E
43	Riversdale – A	RD – A	9	34.31°S – 21.50°E
44	Riversdale – B	RD – B	3	34.23°S – 21.58°E
45	Riversdale – C	RD – C	14	34.10°S – 21.20°E
46	St. Francis – A	SF – A	6	34.17°S – 24.81°E
47	Stellenbosch – A	ST – A	13	33.89°S – 18.89°E
48	Stellenbosch – B	ST – B	14	33.91°S – 18.81°E
49	Stellenbosch – C	ST – C	10	33.85°S – 18.82°E
50	Swellendam – A	SW – A	4	34.05°S – 20.65°E
51	Swellendam – B	SW – B	7	34.40°S – 20.60°E
52	Swellendam – C	SW – C	8	34.19°S – 20.30°E
53	Touwsrivier – A	TR – A	2	33.15°S – 20.47°E
54	Touwsrivier – C	TR – C	5	33.17°S – 20.26°E
55	Worcester – A	WD – A	2	33.59°S – 19.45°E
56	Worcester – B	WD – B	7	33.52°S – 19.49°E
57	Worcester – C	WD – C	10	33.62°S – 19.69°E
58	Beaufort West – A	BW – A	6	32.34°S – 22.64°E
59	Beaufort West – B	BW – B	4	32.34°S – 22.64°E
60	Beaufort West – C	BW – C	2	32.34°S – 22.62°E
61	East London – A	EL – A	3	33.04°S – 27.86°E
62	East London – B	EL – B	1	32.94°S – 27.97°E
63	East London – C	EL – C	2	32.97°S – 27.90°E
64	Graaff-Reinet – A	GR – A	7	32.25°S – 24.53°E
65	Graaff-Reinet – B	GR – B	6	32.17°S – 24.56°E
66	Graaff-Reinet – C	GR – C	8	32.26°S – 24.54°E
67	Klawer – A	KL – A	1	32.02°S – 18.78°E
68	Klawer – B	KL – B	8	32.17°S – 18.51°E
69	Klawer – C	KL – C	6	32.10°S – 18.84°E
70	European *Apis mellifera*	AM	10	
	Total		474	

Bees were sampled from 69 apiaries (no.). The apiaries are identified by their geographic region (the city closest to all apiaries in the region) and apiary in/around that city (A – C sampled apiaries in a region). This was coded into an apiary identifier which includes a two-letter city abbreviation and apiary letter. N represents the number of honey bees examined from each apiary. The GPS location of each apiary is reported in the final column

### Dissection and collection of morphometric data

Lateral images of the thorax and hairs on abdominal tergite 6 (A6) were taken of each bee prior to dissection. Four different parts of each bee’s body (right forewings, right hindwings, tergite A3 and sternite A4) were dissected to facilitate imaging of the morphometric characteristics. Forewings and hindwings were removed from the thorax using forceps. The sternites and tergites were removed by tearing the connective tissue between them. The dissected sternite A4 was cleaned with a paint brush and soaked in KOH to remove any remaining tissue. It also was stained with Bioquip double stain (6379B) and dried on a Kim-tech wipe. Excess stain was removed with ethanol. All body parts were dried on a Kim-tech wipe and mounted on slides using Euparal mounting medium. Mounts were made on 25 × 75 mm Fisher glass slides (S17466A) and cover glass (12–518-105H, Thermo Fisher Co.), warmed at 60 °C for 3 days on a slide warmer (Premiere xh-2004 or C.S. & E. 26,020), and imaged individually using Leica M205 light microscope equipped with a Leica MC170 camera (1600 × 1200 pixels) with its related software.

Ten standard morphometric characters were chosen based on information published by [9, 22]. These characters included: 1) forewing length, 2) the length of cover hair on abdominal tergite A6, 3) transverse width (TW) of the wax plate on abdominal sternite A4, 4) transverse length (TL) of the wax plate on abdominal sternite A4, 5) pigmentation of abdominal tergite A3, 6) number of ovarioles, 7) pigmentation of the scutellum, 8) pigmentation of the scutellar plate, 9) forewing angle N23, and 10) forewing angle O26. The pigmentations of the abdominal tergite A3, scutellum and scutellar plates were determined per the standard color ranks established by Ruttner [4]. Tergites and scutellar plates were ranked from 0 (fully pigmented black) to 9 (no black pigmentation). The scutellum color was ranked from 0 (fully pigmented black) to 5 (no black pigmentation). To avoid bias and improve accuracy, pigmentation was assessed by three different observers for each sample, and the resulting average used for analysis.

**Fig. 2:**
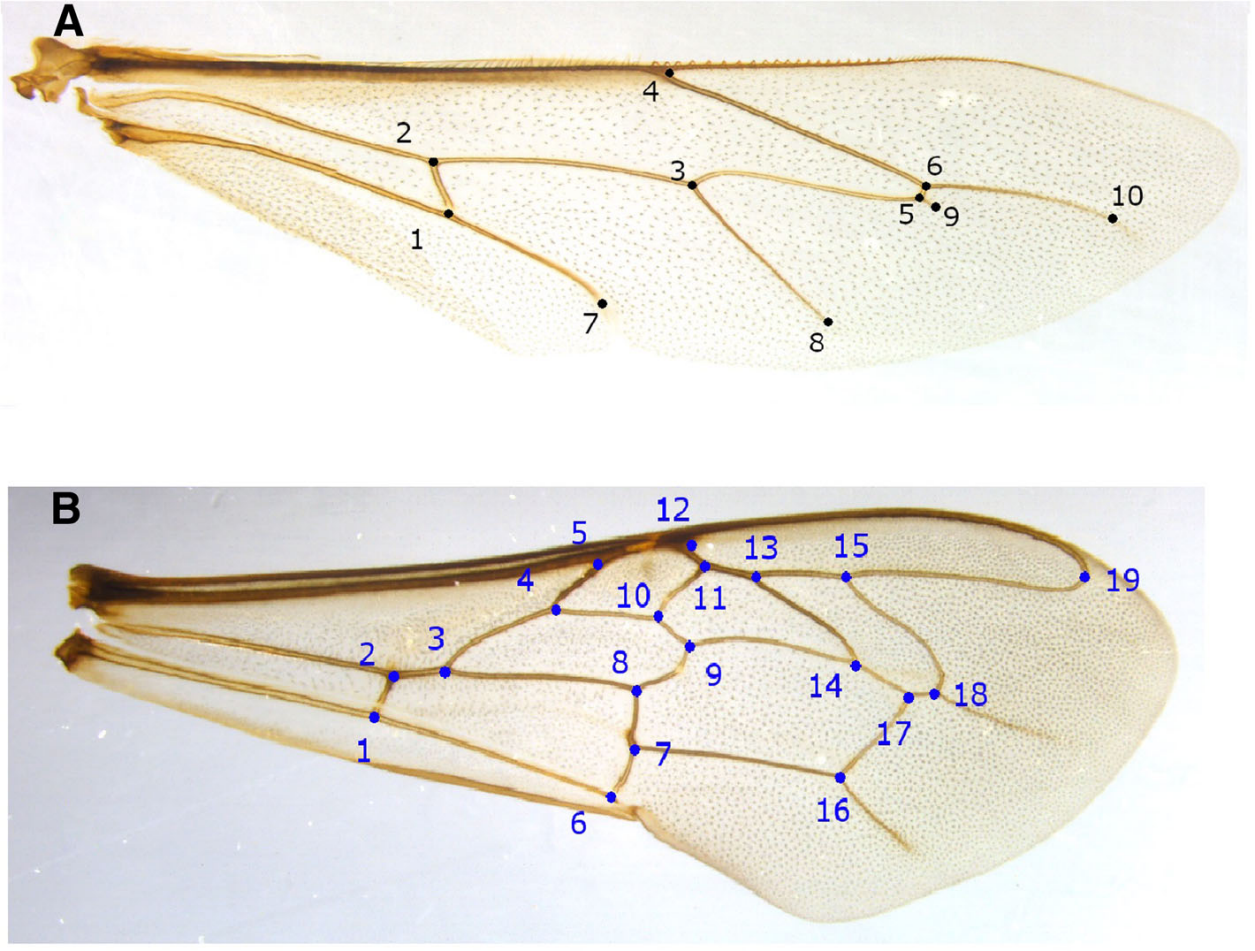
Location of the geometric landmarks on the honey bee wing. **a** 10 landmarks on the right hindwing; **b** 19 landmarks on the right forewing

Forewing geometric landmarks were chosen based on the information published by Francoy et al. [39]. These included 19 landmarks of right forewings (venation intersections). Ten additional landmarks of the right hindwings were also included (Fig. 2). The two-dimensional x, y Cartesian coordinates of the identified landmarks were recorded using custom-built, assistive measuring software (unpublished licensed, software development project by Honey Bee Research and Extension Laboratory, University of Florida).

### DNA extraction

After morphometric analysis, total DNA was extracted from the dissected honey bee thoraces in accordance with the protocol outlined in [40]. DNA quality was assessed using a 1% agarose gel and quantified using Qubit® 3.0 Fluorometer per manufacturer’s guidelines (Thermo Scientific Inc., USA). The extracted DNAs were submitted for sequencing at the Genomic Diversity Facility of Cornell University. The DNA concentration was normalized (< 10 ng/ul) prior to sequencing. Samples with failed extractions were excluded from further analysis.

### Genotyping-by-sequencing (GBS), sequence alignments and quality control

We constructed a GBS library containing 474 honey bee DNA samples (5 × 96 plate), including a negative control (no DNA) in accordance with the methods outlined by [31, 41]. Each DNA sample was digested with methylation-sensitive *Eco*T22I, a type II restriction endonuclease which recognizes a degenerate 6 bp sequence (ATGCAT) (New England Biolabs, Ipswich, MA), by incubation at 37 °C for 2 h. The digested DNAs were ligated with an equal amount of a different barcode-containing adapter and the same common adapter. The 474 barcode sequences were pooled (5 μl each) and purified with QIAquick PCR Purification Kit (Qiagen, Valencia, CA). The sequence of barcodes used for *Eco*T22I GBS library construction was published by [31, 41]. The pooled library was amplified by PCR per the cycling conditions outlined in [31]. The amplified genomic fragments were purified and quantified using a Nanodrop 2000 (Thermo Scientific, Wilmington, DE) based on [31]. The constructed pooled *Eco*T22I library was sequenced on an Illumina HiSeq 2000 (Illumina Inc., San Diego, CA) in one sequencing flowcell lane (100 bp single-end sequencing) at the Cornell University Life Sciences Core Facility.

The raw Illumina DNA sequence reads for the *Eco*T22I library were quality-filtered by removing adapter sequences and enzyme recognition sites, followed by trimming by quality score utilizing the GBS analysis pipeline as implemented in TASSEL v3.0 [42]. We retained only the highest quality first 64 bp of each sequence to minimize the errors associated with base calling. To determine genomic SNP coordinates, we aligned sequence reads to the *A. mellifera* reference genome [43] using the Burrows-Wheeler alignment tool (BWA) version 0.7.8- r455 [44]. We further filtered the resulting genotypes by minor allele frequency (MAF) > 0.01, and missing data per site < 0.1. The filtered *Eco*T22I library reads produced the average individual depth of 38.75 (SD ± 6.76; median 38.61) with the average site depth of 27.88 (SD ±41.8; median 7.3) across all genotypes. All submitted samples generated sufficient genotypes for analysis and the effectiveness of the GBS method using *Eco*T22I digestion genomic library was previously shown by [45].

### Post SNP filtering pipelines

Prior to the population genetic analyses, stringent filtering strategies were performed to obtain the most informative SNPs using VCFtools [46]. We applied eight different filters using the following parameters: (1) minimum read depth > 6 (−-min-meanDP 6), (2) MAF > 5% (−-MAF 0.05), (3) missing data at no more than 5% of samples (−max-missing 0.95), (4) average read depth < 100 (−max-meanDP 100), (5) missingness on per individual (−-missing-indv), (6) remove indel between reads (−-remove-indels), (7) include only bi-allelic sites (−-min-alleles 2 --max-alleles 2), and (8) remove SNPs in linkage disequilibrium (LD) (−-exclude-positions). After applying all filtering pipelines, we identified 2449 biallelic loci for further analysis of the 474 individuals.

### Detecting SNP loci under selection based on *F*_ST_ outlier tests

Two different coalescent-based simulations were used to detect SNP loci deviating from neutrality. With these approaches, we expected to detect low levels of genetic differentiation under balancing selection (neutral loci) and high levels of differentiation under directional selection (divergent loci). We used two different *F*_ST_-based methods including FDIST [47] and hierarchical [48]. FDIST was performed using the program LOGISTAN [49]. LOGISTAN calculates the neutral distribution of *F*_ST_ values with significant *P*-value for each locus. This method computes the distribution between *F*_ST_ and expected heterozygosity (HE) using two options “neutral mean *Fst*” and “force mean *Fst*” to detect genes under selection. A total number of 2449 SNPs were analyzed based on the following parameters: 50,000 simulations, confidence interval of 0.95, false discovery rate of 0.1, attempted *F*_ST_ ≥ 0.9, and mutation model of infinite alleles. We considered the *F*_ST_ values higher than expected neutral distribution as directional selection and *F*_ST_ values lower than expected neutral distribution as balancing selection.

The hierarchical method is a modification of the FDIST approach performed using an Arlequin package ver. 3.5.1.2 [48]. We used a hierarchical island model with 50,000 simulations to calculate the relationships between *F*_ST_ and heterozygosity. Loci with *F*_ST_ values above the 0.99 limits of neutral distribution were considered as putative outliers under the divergent selection [50]. The remaining loci with non-significant *F*_ST_ values were considered as neutral SNPs. All procedures reduced the bias and kept the highly diverged loci between individuals of subspecies.

### Gene ontology (GO) analysis

The closest gene to each of the 83 divergent SNPs was determined using bedops v 2.4.22 in vcf2bed and closest features [51], relative to the gene annotations (gff3 file) which was available from NCBI (https://www.ncbi.nlm.nih.gov/assembly/GCF_000002195.4/). The DAVID gene accession conversion tool was used to identify homologous genes from *Apis mellifera* and *D. melanogaster* as listed in BeeBase and FlyBase [52]. Functional enrichment of these gene IDs was conducted in the GeneMania and g:Profiler platforms [53, 54].

### Environmental data

Nineteen bioclimatic variables were obtained from the WorldClim database (acquired in January 2016 at http://www.worldclim.org/). These bioclimatic variables (Additional file 1: Table S1) at a resolution of 2.5 arc-minutes derived from basic monthly climatic variables generated through interpolation of average monthly climate data from weather stations on a 0.5 arc-minute resolution grid [55]. These derived bioclimatic variables could better reflect biologically meaningful information instead of raw precipitation and temperature variables [56, 57]. We acquired these data for the 69 apiaries using each apiary’s geographic coordinates.

### Statistical analysis

#### Morphometric analysis

The wing images from each bee were scaled, rotated and aligned using a Generalized Procrustes Alignment analysis (GPA) [58]. GPA analysis is a standard method to align landmark coordinate data [59]. Both the wing geometry and standard morphometric data were analyzed to determine which variables can best discriminate the subspecies using linear stepwise discrimination function analysis (a form of multivariate analysis of variance) (JMP ver. 12, SAS Institute, Cary, NC). Cross-validation (JMP ver. 12, SAS Institute, Cary, NC) was applied to determine the cutoff value and confirm accuracy for each wing geometry and standard morphometric parameter. A One-way Analysis of Variance (ANOVA) was applied to determine the average for each wing geometry and standard morphometric parameter (JMP ver. 12, SAS Institute, Cary, NC). A Tukey multiple comparisons test was used to compare mean values of each parameter at α ≤ 0.05). A dendrogram showing the relationships between individuals based on wing shape and morphometric characteristics was made using the unweighted pair group method with arithmetic means (UPGRMA). The analysis was done with 1000 as the bootstrap value and based on the discriminant values of each bee. Our stepwise discrimination function analysis to differentiate subspecies was based on using a combination of wing geometry/standard morphometric data. Once stepwise analysis had determined the best characteristics to use, standard loadings were calculated in R v.3.4.2. The subspecies groups were assigned based on the historical geographical distribution shown by [9]. The northernmost and southernmost bees were considered *A.m. scutellata* and *A.m. capensis* respectively. Bees falling between the two geographical regions were identified as hybrids. We repeated the analysis, grouping together samples by region instead of subspecies.

#### Population genetics analyses

We computed the pairwise evolutionary divergence among regions using MEGA7 [60] based on the *p*-distance model with 1000 bootstrap value across entire SNP loci. Population genetic diversity indices such as observed heterozygosity (*H*_obs_), expected heterozygosity (*H*_exp_), proportion of polymorphic SNP (*N*_p_), and inbreeding coefficient (*F*_IS_) were calculated for SNP loci using the statistical package R. Departure from Hardy-Weinberg equilibrium (HWE) was assessed by an exact test using Genepop 4.2 [61] based on the following Markov chain Monte Carlo simulation parameters: dememorization = 5000, batches = 5000, and iterations per batch = 1000 [62]. Analysis of molecular variance (AMOVA) was performed to determine the proportion of genetic variation within and between regions as implemented in Arlequin 3.5.1.2 [48] with 1000 permutations. The populations were structured by aligning Bayesian clustering pattern with the historical geographic distribution of the bees [9]. The overall and pairwise values of population differentiation statistic (*F*_ST_) [63] were determined among regions and within subspecies using the SNP loci as calculated by Arlequin ver. 3.5.1.2 [48]. We permuted 1000 iterations to calculate the *p*-values for the mean and pairwise *F*_ST_ values. *F*_ST_ varies from 0 (lack of genetic structure and no sign of population subdivision) to 1 (distinct population structure or extreme segregation), with *F*_ST_ of up to 0.05 indicating a moderate genetic differentiation [64].

#### Association of environmental variables with SNP loci

We examined the association of divergent SNP loci with environmental characteristics using an individual-based spatial analysis as implemented in Samβada program [65] (available at lasig.epfl.ch/sambada). Samβada examines the associations between all environmental variables and allele frequencies of divergent SNP loci across sampling locations by a logistic regression approach. Models are selected through the examination of the significance of regression coefficient across environmental variables. The significance value of each model was evaluated with likelihood G and Wald scores. The Bonferroni-corrected threshold was considered as α = 0.05, indicating a significant association between loci and the environmental variables.

#### Bayesian population structure and principal coordinates analysis (PCoA)

We applied a model-based Bayesian clustering approach to characterize the existence of distinct genetic clusters among regions of both subspecies as implemented in STRUCTURE 2.3.4 [66]. The genome of each bee positioned into a predefined number of components (*K*) with variable proportions of allele frequency of the ancestral population. This approach allows the characterization of an ancestral population using admixed bees [66]. We ran STRUCTURE for *n* = 2449 loci and q subset of divergent loci (*n* = 83) using an admixture model and by applying a putative number of clusters (*K*) varying from 1 to 10. The analysis was performed without prior information of population identity by a simulation of 50,000 pre-burn steps and 100,000 post-burn iterations of MCMC algorithm for each run. We performed 10 independent runs for each *K* to estimate the most reliable number of distinct genetic clusters (*K*) using the likelihood of the posterior probability (LnP (N/K)) [67] and ad hoc quantity DK for each *K* partition. Posterior probability changes with respect to *K* between different runs are assigned as a method for determination of the true *K* value [68]. The most likely value for *K* was identified based on average log likelihood, Ln P (D) using Evanno’s ∆K method [68] from the web-based software STRUCTURE HARVESTER [69]. The population structure barplots were visualized using the CLUMPAK program as implemented in CLUMPP ver. 1.1.2 [70].

A principal coordinate analysis (PCoA) was performed with both sets of SNPs and the entire and divergent SNP loci to visualize the divergence pattern among individuals between two subspecies using TASSEL v3.0 and JMP Pro v12 (SAS Institute, Cary, NC). The PCoA analysis enables us to visualize the relatedness of individual honey bees across individuals/regions in multidimension scales.

## Results

### Wing geometry and standard morphometrics

The 19 forewing landmarks created 38 Cartesian coordinates for each specimen and the ten hindwing landmarks generated 20 Cartesian coordinates for each specimen. At the subspecies level, the linear stepwise discriminant function analysis of combined wing geometry and standard morphometric variables incorporated six out of ten variables (ovariole number, abdomen hair, scutellar plate, angle O, forewing length and tergite color), 11 out of 38 forewing coordinates (F4X, F5X, F6X, F6Y, F7X, F8X, F15Y, F16X, F16Y, F18Y, F19Y), and ten out of 20 hindwing coordinates (H2X, H2Y, H3Y, H5X, H7X, H7Y, H8X, H9X, H9Y, H10Y) in the statistically significant classification model for the honey bee populations (*P* < 0.05). The linear stepwise discriminant function analysis based on all examined variables placed all individual bees into the three groups with a high percentage of classification (87%). The cross-validation test misclassified 61 (13.11%) out of 464 bees. At the regional level, a linear stepwise discriminant function analysis of all wing geometry and standard morphometric variables included six out of ten standard variables (ovariole number, abdomen hair, scutellar plate, scutellum, sternite H, angle N, forewing length and tergite color), 12 out of 38 forewing coordinates (F1Y, F2Y, F4Y, F6Y, F7Y, F8Y, F10Y, F12Y, F13Y, F16Y, F18Y, F19Y), and seven out of 20 hindwing coordinates (H1X, H1Y, H2X, H2Y, H3Y, H10X, H10Y) in the significant classification model for the honey bee populations (*P* < 0.05). A linear stepwise discriminant function analysis based on all examined variables placed all individual bees into the three groups with an average percentage of classification (53%). The cross- validation test misclassified 217 (47%) out of 464 bees.

**Fig. 3:**
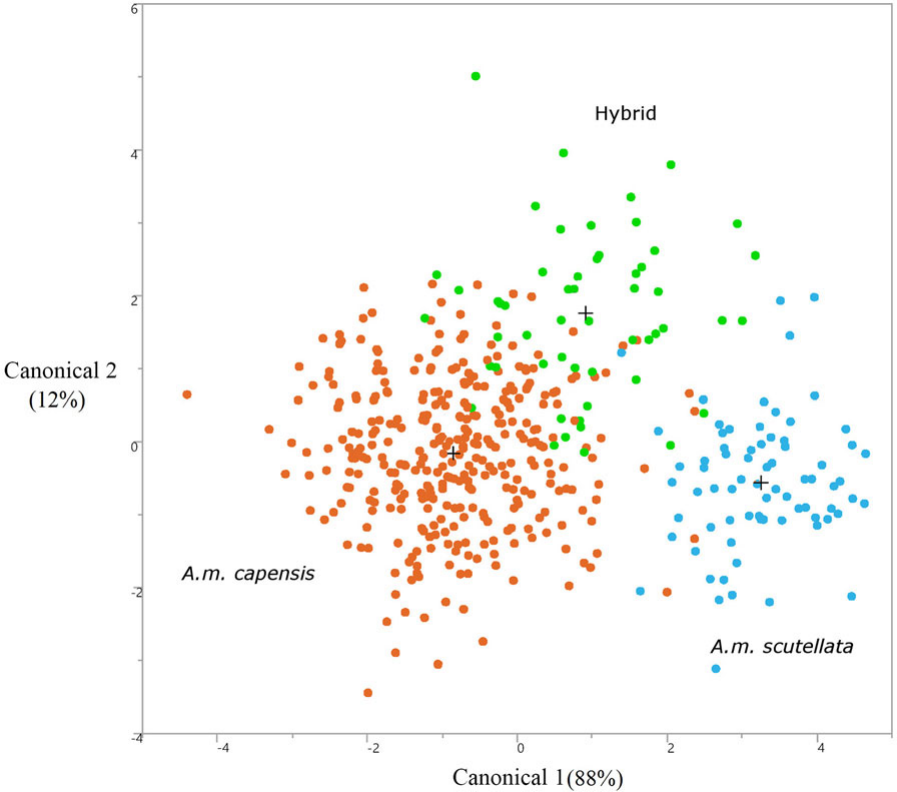
Morphological clustering pattern based on the final model from a stepwise discriminant analysis using 48 wing geometry and standard morphometric parameters. Three distinct morphological groups are shown by different colors, of which orange (*N* = 337), blue (*N* = 73) and green (*N* = 54) colors reflect *Apis mellifera capensis*, *A.m. scutellata* and hybrid honey bees respectively

The discriminant function analysis of all 464 honey bees showed that the clusters created by the three groups overlapped in Canonical space. Canonical Vector 1 (CV1) explained 88% of the variance and Canonical Vector 2 (CV2) explained 12% (Fig. 3). Twenty-seven of 39 wing geometry and standard morphometric parameters have variations in the positive and negative factor-loading axis onto CV1 after normalizing the data. The ovariole count (− 0.45) and scutellar plate color (− 0.42) were significant characters in the first canonical function and the hindwing landmark coordinate 7X (0.59) and forewing coordinate 7X (0.55) in second canonical function. The least influential characters were the forewing landmark coordinate 7X (− 0.02) and hindwing coordinate 2X (0.03) in the first canonical function, and forewing coordinate Y15 (− 0.03) and forewing length (− 0.03) in the second canonical function (Additional file 2: Figure S1).

An analysis of the honey bees grouped into the 28 geographical regions where they were collected revealed an incongruent and overlapped clustering pattern in the Canonical scatter plot of CV 1 (33%) and CV 2 (12%). The ovariole count (0.54) and abdomen hair length (− 0.47) were significant characteristics in the first canonical function and the forewing landmark coordinate 5X (0.76) and forewing angle O (0.51) in the second canonical function. The least influential characteristics were forewing coordinate X7 (0.007) and hindwing coordinate X2 (0.007) in the first canonical function, and forewing coordinate X7 (0.03) in the second canonical function (Additional file 3: Figure S2).

A dendrogram constructed by hierarchical cluster analysis of the squared Euclidian distances across all individuals revealed six major morphological groups (Additional file 4: Figure S3). Each group was composed of bees clustering across all three major groups (*A.m. capensis*, *A.m. scutellate* and hybrids). The cluster analysis showed a consistent pattern of overlap with hybrids located between the two subspecies.

The mean values of wing geometry and standard morphometric variables for three groups (*A.m. scutellata*, *A.m. capensis* and hybrids) are presented in Table 2. *F* values generated significant differences among all three groups except for characteristics 4, 7, 8, 11, 12, 14, 20, 21, 22, 24 and 25 as depicted in Table 2. Scutellar plate and tergite color show the highest between-group variability and statistical differences among mean values (Table 2). In contrast hindwing characteristic 5X, hindwing characteristic 9X, hindwing characteristic 8X, and forewing characteristic 16X have the lowest variability and are not significantly different from other parameters (Table 2).

**Table 2:** The mean values (SD) of wing geometry and standard morphometric characters calculated for *Apis mellifera capensis, A.m. scutellata*, and hybrids of the two

Characters	*A.m. scutellata*	*A.m. capensis*	Hybrid	*F*-value	*P*-value
number of ovarioles	5.12^a^ (0.69)	12.76^c^ (0.32)	9.24^b^ (0.8)	53.61	< 0.01
abdomen hair (mm)	0.0002^a^ (0.00)	0.0001^c^ (0.00)	0.0001^b^ (0.00)	84.09	< 0.01
scutellar plate (0–9 scale)	7.86^a^ (0.29)	2.42^c^ (0.13)	5.01^b^ (0.34)	149.57	< 0.01
angle O (degrees)	1.01^a^ (0.007)	1^a^ (0.003)	1^a^ (0.008)	0.21	0.81
forewing length (mm)	0.008^a^ (0.00)	0.008^b^ (0.00)	0.008^b^ (0.00)	13.73	< 0.01
tergite color (0–9 scale)	8.45^a^ (0.18)	5.37^c^ (0.08)	6.94^b^ (0.21)	129.62	< 0.01
forewing 4X	705.38^a^ (5.18)	716.41^a^ (2.4)	706.22^a^ (6.02)	2.69	0.06
forewing 5X	749.32^a^ (5.44)	758.56^a^ (2.52)	748.79^a^ (6.32)	1.91	0.14
forewing 6X	775.10^b^ (4.99)	788.60^a^ (2.32)	786.57^ab^ (5.81)	3	0.05
forewing 6Y	655.17^b^ (7.53)	703.39^a^ (3.5)	688.55^a^ (8.75)	17.08	< 0.01
forewing 7X	794.09^a^ (4.98)	806.24^a^ (2.31)	803.11^a^ (5.8)	2.45	0.08
forewing 8X	797.61^a^ (5.01)	808.47^a^ (2.32)	802.33^a^ (5.82)	2.16	0.11
forewing 15Y	445.63^b^ (7.03)	491.01^a^ (3.26)	468.22^b^ (8.17)	18.56	< 0.01
forewing 16X	1009.21^a^ (4.97)	1015.71^a^ (2.31)	1015.14^a^ (5.78)	0.71	0.49
forewing 16Y	629.11^b^ (7.03)	669.71^a^ (3.26)	649.61^ab^ (8.18)	14.79	< 0.01
forewing 18Y	547.41^b^ (6.97)	587.36^a^ (3.24)	564.68^b^ (8.11)	15.13	< 0.01
forewing 19Y	437.97^b^ (7.07)	477.39^a^ (3.29)	450.20^b^ (8.23)	15.41	< 0.01
hindwing 2X	561.13^ab^ (4.77)	570.35^a^ (2.22)	554.38^b^ (5.55)	4.47	0.01
hindwing 2Y	506.04^b^ (7.2)	546.63^a^ (3.34)	557.79^a^ (8.37)	15.19	< 0.01
hindwing 3X	852.26^a^ (4.85)	857.30^a^ (2.25)	848.77^a^ (5.63)	1.25	0.28
hindwing 5X	1096.02^a^ (4.98)	1096.60^a^ (2.31)	1092.79^a^ (5.79)	0.19	0.83
hindwing 7X	734.21^a^ (5.51)	745.44^a^ (2.56)	736.75^a^ (6.4)	2.19	0.11
hindwing 7Y	664.52^b^ (7.14)	701.16^a^ (3.32)	709.20^a^ (8.31)	12.16	< 0.01
hindwing 8X	1003.83^a^ (5.04)	1008.5^a^ (2.34)	1005.01^a^ (5.86)	0.43	0.64
hindwing 9X	1104.13^a^ (4.95)	1103.73^a^ (2.3)	1098.92^a^ (5.76)	0.32	0.72
hindwing 9Y	549.09^b^ (7.13)	585.29^a^ (3.31)	589.01^a^ (8.29)	11.25	< 0.01
hindwing 10Y	581.94^b^ (7.19)	616.71^a^ (3.34)	614.33^a^ (8.36)	9.7	< 0.01

Row means with the same superscript letters are not significantly different from one another (Tukey’s test, *P* ≤ 0.05

At the regional level, forewing characteristics 1Y (*F* = 6.84), 2Y (*F* = 6.9) and 4Y (*F* = 6.39) show the highest between group variability and statistical difference among mean values (*P* ≤ 0.05), while angle N (*F* = 1.85) and hindwing characteristic 10X (*F* = 1.31) have the lowest variability and are not significantly different from other parameters (*P* > 0.05) (data not shown).

### Genotypic data, genetic diversity and divergence

A total of 3,103,730 reads of paired-end sequencing data were generated with 474 individual bees using the GBS method. An average of 65% were uniquely aligned to the honey bee reference genome (GCF_000002195.4_Amel_4.5_genomic.fna.gz), resulting in 2,028,130 reads with 98,134 SNPs. After evaluating the dataset with informative pipelines (MAF overall populations > 0.01, missing data per site < 90%), we filtered 70,475 SNPs with a mean individual depth of 38.7. By applying stringent additional post filtering SNP criteria, we kept 2449 high quality SNPs out of 70,475 total to analyze among the 474 individual bees in the final data set. A test for HWE departure indicated that all SNPs and regions are in HWE after the sequential Bonferoni correction. At the regional level, ten of 29 regions were in HWE (BL, CD, GE, GR, KN, LA, PE, RD, ST and SW – see Table 1 for abbreviation location) but the rest showed a significant departure from HWE. Genetic diversity estimates for mean value of allelic richness for *A.m. capensis*, *A.m. scutellata* and hybrids were 1.53, 1.52 and 1.52 respectively. A similar level of mean observed and expected heterozygosity was found for *A.m. capensis*, *A.m. scutellata*, and hybrids (Table 3). The observed heterozygosity (*H*_obs_) was the highest across all regions (*H*_obs_ = 0.26) except for the PT region (*H*_obs_ = 0.21). The inbreeding coefficient (*F*_IS_) was negative in BD, MF, OD and TR regions for *A.m. capensis* and in the KR region for *A.m. scutellata*. Within the hybrids, two regions, BW and KL, generated negative values that indicate an outbreeding outcome in these regions. The *F*_IS_ value estimated for the 2449 SNP loci revealed the inbreeding outcome occurred across the regions. The percentage of polymorphic SNPs (*N*_p_) ranged from 60.27 to 95.51% among regions. The mean percent of polymorphic SNPs was higher in *A.m. scutellata* than in *A.m. capensis* and hybrid populations (Table 3). The population genetic diversity indices across 28 geographical regions is depicted in Table 3. Fifteen regions of *A.m. capensis*, two regions of *A.m. scutellata* and two regions of hybrids showed the maximum amount of genetic divergence (0.08). The minimum value was observed in two regions of *A.m. capensis* (BD and TR), and one region of *A.m. scutellata* (KR) (0.06). A net pair-wise evolutionary divergence among regions ranged from 0.01 to 0.08, with an average of 0.09 (Additional file 5: Table S2).

**Table 3:** Population genetic characteristics, determined using 2449 SNP loci, of the honey bees sampled from 28 geographical regions in the Republic of South Africa

Geographic region	*N*_a_	Ar	*H*_obs_	*H*_exp_	*F*_IS_	*N*_p_ (%)
*Apis mellifera scutellata, N* = 73 bees						
BL	1.88	1.52	0.24	0.23	0.04	88.18
KR	1.87	1.53	0.25	0.23	−0.02	87.03
PT	1.71	1.48	0.21	0.21	0.003	71.8
SP	1.73	1.51	0.22	0.22	0.001	73.81
UP	1.88	1.54	0.25	0.23	0.01	88.1
Mean	1.81	1.52	0.24	0.22	0.006	87.78
*Apis mellifera capensis*, *N* = 337 bees						
BD	1.6	1.45	0.22	0.19	−0.04	60.27
CD	1.93	1.55	0.24	0.24	0.02	93.12
CT	1.92	1.55	0.25	0.24	0.03	92.46
GE	1.94	1.55	0.23	0.24	0.06	94.48
GT	1.86	1.53	0.23	0.23	0.02	87
KN	1.95	1.55	0.24	0.24	0.04	95
LA	1.92	1.55	0.23	0.24	0.04	92.26
LB	1.82	1.54	0.24	0.23	0.02	82.37
MB	1.72	1.52	0.23	0.22	0.003	72.33
MF	1.86	1.55	0.26	0.24	−0.01	86.66
OD	1.76	1.52	0.23	0.22	− 0.02	76.5
PB	1.85	1.53	0.24	0.23	0.02	85.6
PE	1.9	1.54	0.23	0.24	0.03	90.4
RD	1.94	1.55	0.24	0.24	0.04	94.03
SF	1.69	1.5	0.23	0.22	0.03	69
ST	1.95	1.54	0.23	0.24	0.04	95.51
SW	1.87	1.53	0.24	0.23	0.03	87.64
TR	1.66	1.48	0.23	0.21	−0.04	66.07
WD	1.91	1.54	0.25	0.23	0.03	91.23
Mean	1.84	1.53	0.24	0.23	0.02	84.83
Hybrid bees, *N* = 54 bees						
BW	1.81	1.52	0.25	0.23	−0.02	81.22
EL	1.69	1.5	0.23	0.21	0.02	69.41
GR	1.9	1.54	0.23	0.23	0.02	90.77
KL	1.83	1.52	0.24	0.23	−0.001	83.07
Mean	1.81	1.52	0.24	0.22	0.004	81.12
European *A. mellifera* reference, *N* = 10 bees						
AM	1.36	1.25	0.12	0.11	−0.05	35.73

Geographical abbreviations are defined in Table 1. Abbreviations: *N*_a_, mean number of observed alleles per locus; *Ar*, mean allelic richness; *H*_obs_, observed heterozygosity; *H*_exp_, expected heterozygosity; *F*_IS_; Fixation index and *N*_P_ (%), percent mean number of private alleles per region. Mean values are calculated by pooling correspondent regions of each subspecies

### Detection of divergent SNP loci

**Fig. 4a, b:**
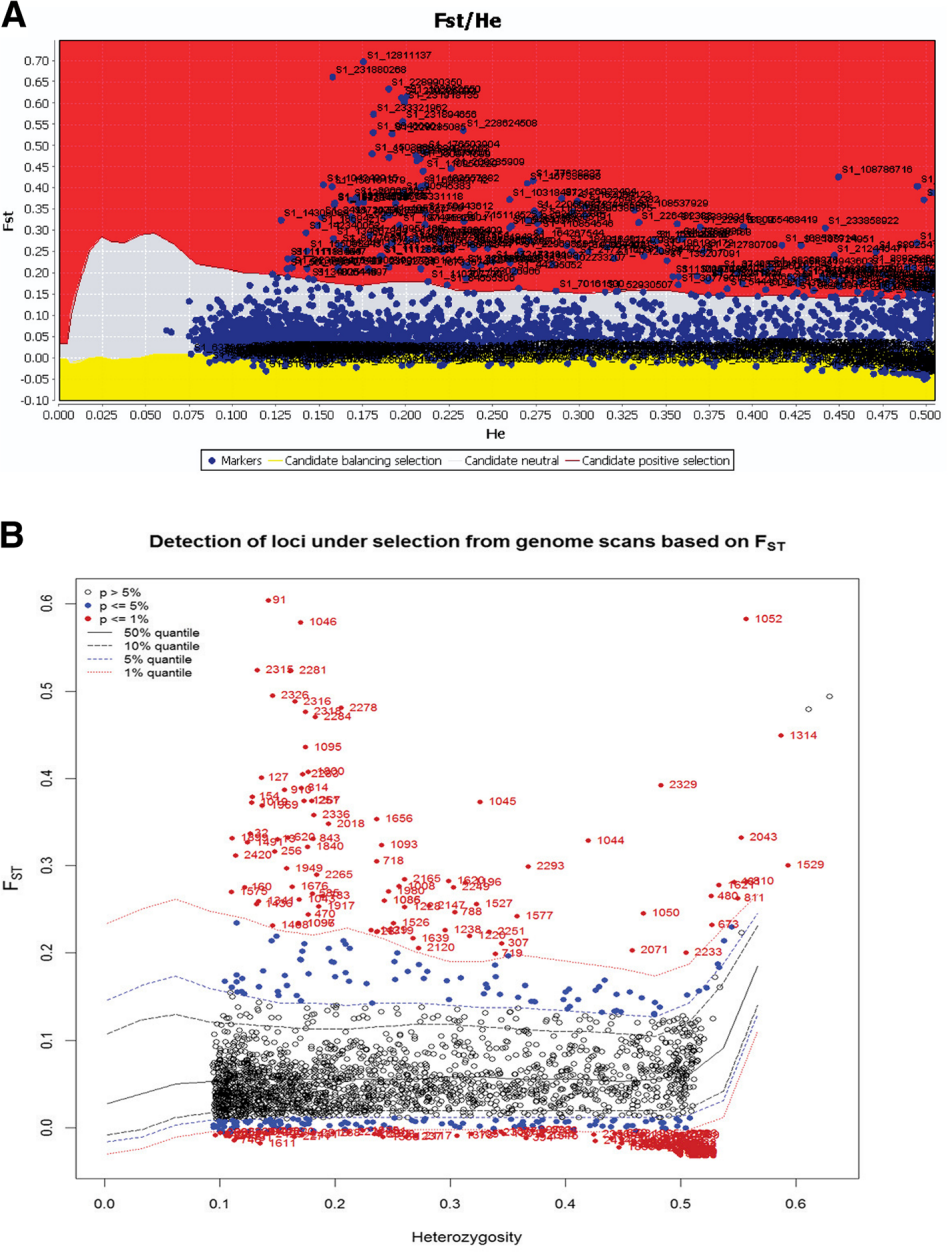
Identification of putative divergent SNP loci under directional selection based on *F*_ST_ outlier approaches. **a** Hierarchical structure model using Arlequin 3.5. *F*_ST_: locus –specific genetic divergence among the populations; Heterozygosity: measure of heterozygosity per locus. The significant loci are shown with red dots (*P* < 0.01). **b** Finite island model (fdist) by LOGISTAN. Loci under positive selection above 99% percentile are shown in the red area. Loci in the gray area are neutral loci. Those in the yellow area are under balancing selection

When we considered all 474 honey bees including *A.m. scutellate*, *A.m. capensis* and the European *A. mellifera*, the Arlequin hierarchical method revealed 90 candidate SNPs for divergent selection at the 1% significance level (Fig. 4a). Based on the same data set, the LOGISTAN method detected 103 divergent loci with evidence of divergent selection (4b). These approaches concurred on 83 divergent SNPs at the α= 0.01 significance level. The list of these 83 divergent SNP loci, together with their locations in the honey bee genome and information on functional gene annotation, are listed in Table 4.

**Table 4:** List of 83 divergent SNPs with their functions obtained by outlier tests as putative markers and used for their association with environmental variables using Samβada package

SNPID	SNP Position	Gene product	Gene ID (BEEBASE)	Gene ID (FLYBASE)
S1_4,550,788	4,550,788	LIM/homeobox protein Awh	GB53183	FBgn0013751
S1_6,686,923	6,686,923	Uncharacterized LOC725682, transcript variant X1	GB40830	FBgn0260997
S1_12,811,137	12,811,137	Protein prickle-like, transcript variant X1	GB44796	FBgn0003090
S1_15,039,694	15,039,694	Krueppel-like factor 6, transcript variant X1	GB52133	FBgn0040765
S1_17,699,574	17,699,574	Protein-serine O-palmitoleoyltransferase porcupine	GB47458	FBgn0004957
S1_18,694,215	18,694,215	Protein eva-1, transcript variant X1	GB51947	FBgn0259821
S1_33386163	3,492,655	Unknown	Unknown	Unknown
S1_50443612	5,000,737	Dumpy	GB55781	FBgn0053196
S1_52257404	6,814,529	Unknown	Unknown	Unknown
S1_52257424	6,814,549	Unknown	Unknown	Unknown
S1_62957554	4,280,238	Sestrin homolog, transcript variant X1	GB49567	FBgn0034897
S1_69083742	10,406,426	Protein 4.1 homolog	GB44175	FBgn0010434
S1_74943997	3,548,247	Hemicentin-2, transcript variant X1	GB51391	Unknown
S1_77638337	6,242,587	Solute carrier family 35 member F5, transcript variant X1	GB46790	FBgn0034032
S1_77638361	6,242,611	Solute carrier family 35 member F5, transcript variant X1	GB46790	FBgn0034032
S1_86109011	349,889	Eye-specific diacylglycerol kinase, transcript variant X1	GB51219	FBgn0261549
S1_86386875	627,753	Lateral signaling target protein 2 homolog	GB51210	FBgn0039492
S1_88925416	3,166,294	Charged multivesicular body protein 7	GB54484	FBgn0027565
S1_88925462	3,166,340	UDP-glucuronosyltransferase 1–3-like	GB54485	Unknown
S1_89224631	3,465,509	Jazigo, transcript variant X1	GB43054	FBgn0261259
S1_90545383	4,786,261	GRAM domain-containing protein 3-like, transcript variant X1	GB52157	Unknown
S1_96460901	10,701,779	Guanylate cyclase, soluble, beta 1	GB52953	FBgn0013973
S1_103184872	17,425,750	Potassium channel subfamily T member 2	GB45474	FBgn0261698
S1_104349915	117,756	Zinc finger protein GLI4-like, transcript variant X1	GB40273	FBgn0039039
S1_108444799	4,212,640	Protein tincar, transcript variant X1	GB49246	FBgn0261649
S1_108537914	4,305,755	Tachykinin	GB49248	Unknown
S1_108537929	4,305,770	Tachykinin	GB49248	Unknown
S1_108682550	4,450,391	Unknown	Unknown	Unknown
S1_108724951	4,492,792	Uncharacterized LOC725260	GB54634	FBgn0034808
S1_108786716	4,554,557	Uncharacterized LOC725260	GB54634	FBgn0034808
S1_110307882	6,075,723	Thyrotropin-releasing hormone-degrading ectoenzyme-like, transcript variant X1	GB43314	Unknown
S1_110854546	6,622,387	CCR4-NOT transcription complex subunit 6-like, transcript variant X1	GB48300	FBgn0011725
S1_110950229	6,718,070	CCR4-NOT transcription complex subunit 6-like, transcript variant X1	GB48300	FBgn0011725
S1_110951124	6,718,965	CCR4-NOT transcription complex subunit 6-like, transcript variant X1	GB48300	FBgn0011725
S1_110951168	6,719,009	CCR4-NOT transcription complex subunit 6-like, transcript variant X1	GB48300	FBgn0011725
S1_123838315	6,386,711	Plasma membrane calcium-transporting ATPase 2	GB43909	FBgn0259214
S1_125133095	7,681,491	Uncharacterized LOC411277, transcript variant X1	GB41894	FBgn0023531
S1_126922494	9,470,890	Probable G-protein coupled receptor 158	GB52840	FBgn0085401
S1_127479850	10,028,246	Ecdysone receptor, transcript variant A	GB48059	FBgn0000546
S1_130971599	13,519,995	Uncharacterized LOC725485	GB51646	FBgn0259927
S1_132727867	1,729,619	Prefoldin subunit 5	GB43750	FBgn0038976
S1_134667854	3,669,606	Fasciclin-1	Unknown	Unknown
S1_141112580	10,114,332	Sortilin-related receptor	GB53341	Unknown
S1_143133972	1,015,171	Uncharacterized LOC102654435	Unknown	Unknown
S1_143743719	1,624,918	Unknown	Unknown	Unknown
S1_150101379	7,982,578	Beta-1-syntrophin, transcript variant X1	GB54295	FBgn0037130
S1_153385191	11,266,390	Uncharacterized protein CG43867	GB51041	FBgn0259100
S1_153549815	11,431,014	Brachyury protein, transcript variant X1	GB51013	FBgn0011723
S1_153549857	11,431,056	Brachyury protein, transcript variant X1	GB51013	FBgn0011723
S1_155093443	8589	Unknown	Unknown	Unknown
S1_155468419	383,565	Uncharacterized protein C1orf112 homolog	GB46620	FBgn0050424
S1_162084123	6,999,269	Protein FAM49B, transcript variant X1	GB53506	FBgn0052066
S1_162216249	7,131,395	tRNA dimethylallyltransferase, mitochondrial-like	GB53505	Unknown
S1_164267544	9,182,690	Carbonic anhydrase-related protein 10, transcript variant X1	GB45092	FBgn0029962
S1_167336698	12,251,844	Tyrosine-protein kinase transmembrane receptor Ror2, transcript variant X1	GB45194	FBgn0020391
S1_168107630	13,022,776	Fringe glycosyltransferase	GB44913	FBgn0011591
S1_176503904	6,692,394	Disks large 1 tumor suppressor protein	GB40648	FBgn0001624
S1_182557882	843,618	Unknown	Unknown	Unknown
S1_192304686	301,823	Inactive rhomboid protein 1	GB49046	FBgn0041723
S1_193370056	1,367,193	Unknown	GB53054	Unknown
S1_197495828	5,492,965	Negative elongation factor E-like, transcript variant X1	Unknown	Unknown
S1_200092025	8,089,162	Chromatin assembly factor 1 subunit A-B, transcript variant X1	GB52767	FBgn0025833
S1_202452286	195,668	MAX dimerization protein	Unknown	Unknown
S1_209285909	7,029,291	Uncharacterized LOC100576529	GB50066	Unknown
S1_212445471	21,524	Scavenger receptor class B member 1-like, transcript variant X1	Unknown	Unknown
S1_217211608	4,787,661	Unknown	Unknown	Unknown
S1_218446595	6,022,648	Ankyrin repeat domain-containing protein 39-like, transcript variant X1	GB45961	FBgn0031674
S1_220569872	317,721	Histone-lysine N-methyltransferase SETMAR-like	Unknown	Unknown
S1_226482382	27,314	Histone-lysine N-methyltransferase SETMAR-like	Unknown	Unknown
S1_226482388	27,320	Histone-lysine N-methyltransferase SETMAR-like	Unknown	Unknown
S1_227605662	117,306	Unknown	Unknown	Unknown
S1_228624508	85,274	Unknown	Unknown	Unknown
S1_228990350	24,441	Unknown	Unknown	Unknown
S1_229285085	47,268	Cytohesin-1-like	GB55123	FBgn0086779
S1_229285093	47,276	Cytohesin-1-like	GB55123	FBgn0086779
S1_229818309	42,135	Unknown	Unknown	Unknown
S1_231880268	35,546	Lactosylceramide 4-alpha-galactosyltransferase, transcript variant X1	GB46832	FBgn0039378
S1_231894656	49,934	Unknown	Unknown	Unknown
S1_231918135	9237	Putative phosphatidate phosphatase, transcript variant X1	GB49207	FBgn0016078
S1_233321962	34,497	Homogentisate 1,2-dioxygenase	GB53288	FBgn0040211
S1_233858922	67,499	Zinc finger and BTB domain-containing protein 24-like	GB51241	Unknown
S1_234352063	52,465	Unknown	Unknown	Unknown
S1_241871073	419	Unknown	Unknown	Unknown

The Gene IDs mapped with *Apis mellifera* and *Drosophila melanogaster* genomes are listed for each SNP

In order to examine environmental correlations within subspecies, we also tested loci under selection within each of the sub-populations. Using the 2449 SNPs within *A.m. capensis*, the Arlequin hierarchical method revealed 54 divergent SNPs (α = 0.01) and LOGISTAN identified 132 divergent SNPs (Additional file 6: Figure S4 A, B). Both approaches revealed 47 divergent SNPs under directional selection (Table 5). For *A.m. scutellata*, the Arlequin hierarchical method revealed 31 divergent SNPs (α= 0.01) and LOGISTAN identified 45 divergent SNPs (Additional file 7: Figure S5 A, B) with 21 divergent SNPs in common between the two approaches (Table 6).

**Table 5:** The list of divergent SNP loci for *Apis mellifera capensis* using *F*_ST_ outlier and environmental correlation tests

No.	SNP loci	SNP Position	Heterozygosity	*F*_ST_ statistic	Environmental correlation
1	S1_13,998,280	13,998,280	0.25	0.1	V1, V6, V10, V11
2	S1_14,309,089	14,309,089	0.13	0.32	
3	S1_19,198,024	19,198,024	0.1	0.13	
4	S1_33680990	3,787,482	0.19	0.26	
5	S1_34025116	4,131,608	0.14	0.18	
6	S1_38187276	8,293,768	0.24	0.11	
7	S1_39180700	9,287,192	0.25	0.11	V15
8	S1_47120892	1,678,017	0.47	0.16	V13, V16, V19
9	S1_50443500	5,000,625	0.39	0.2	
10	S1_52257404	6,814,529	0.46	0.21	V8, V15, V19, Longitude
11	S1_52257424	6,814,549	0.5	0.27	V15, Longitude
12	S1_58794304	116,988	0.12	0.19	
13	S1_71833698	437,948	0.5	0.2	
14	S1_72542859	1,147,109	0.2	0.13	
15	S1_84555306	13,159,556	0.22	0.17	V13, V16, V19
16	S1_84555310	13,159,560	0.21	0.13	V15, Latitude
17	S1_94849186	9,090,064	0.32	0.15	
18	S1_96460917	10,701,795	0.42	0.15	
19	S1_100472193	14,713,071	0.29	0.13	
20	S1_105727753	1,495,594	0.13	0.11	
21	S1_108033805	3,801,646	0.29	0.11	
22	S1_108684700	4,452,541	0.14	0.16	V4, V5, V7, V14, V15, V17, V18, Longitude
23	S1_108684756	4,452,597	0.15	0.16	V4, V5, V7, V14, V15, V17, V18, Longitude
24	S1_108786716	4,554,557	0.45	0.42	V9
25	S1_109332327	5,100,168	0.21	0.25	V3, V4, V5, V7,V14, V15, V17, V18, Longitude
26	S1_110951124	6,718,965	0.18	0.28	V4, V5, V7, V14, V17, V18, Longitude
27	S1_110951168	6,719,009	0.18	0.28	V4, V5, V7, V14, V17, V18, Longitude
28	S1_110951197	6,719,038	0.19	0.27	V3, V4, V5, V7, V14, V15, V17, V18, Longitude
29	S1_111171339	6,939,180	0.18	0.24	V3, V4, V5, V7, V14, V15, V17, V18, Longitude
30	S1_111171425	6,939,266	0.18	0.24	V3, V4, V5, V7, V14, V15, V17, V18, Longitude
31	S1_111285658	7,053,499	0.18	0.23	V4, V14, V15, V17, V18, Longitude
32	S1_118855642	1,404,038	0.48	0.14	
33	S1_120132551	2,680,947	0.2	0.13	Longitude
34	S1_132727867	1,729,619	0.2	0.25	V3, V9, Longitude
35	S1_132761070	1,762,822	0.5	0.38	V3, V9, Longitude
36	S1_133026966	2,028,718	0.23	0.18	Longitude
37	S1_146906109	4,787,308	0.16	0.21	
38	S1_152157525	10,038,724	0.43	0.14	
39	S1_158767923	3,683,069	0.44	0.11	
40	S1_177861915	8,050,405	0.2	0.2	V1, V5, V8, V10
41	S1_192304686	301,823	0.16	0.36	V8
42	S1_207842400	5,585,782	0.07	0.13	
43	S1_210484669	8,228,051	0.3	0.1	
44	S1_212486639	62,692	0.5	0.17	Longitude
45	S1_238491065	11,486	0.16	0.15	
46	S1_238491098	11,519	0.36	0.15	V13, V15, V16, V19
47	S1_238498932	1677	0.47	0.11	

The significant SNP genotypes correlated with environmental variables for both the likelihood ratio (G) and Wald tests at the Bonferroni-corrected 0.05 alpha level are shown. The variable numbers are explained in Additional file 1: Table S1

**Table 6:** The list of divergent SNP loci for *Apis mellifera scutellata* using *F*_ST_ outlier and environmental correlation tests

No.	SNP loci	SNP Position	Heterozygosity	*F*_ST_ statistic	Environmental correlation
1	S1_22,206,418	22,206,418	0.21	0.1	
2	S1_39180699	9,287,191	0.1	0.17	
3	S1_75314820	3,919,070	0.17	0.08	
4	S1_88966222	3,207,100	0.48	0.1	Latitude
5	S1_89371176	3,612,054	0.3	0.11	
6	S1_90412686	4,653,564	0.13	0.12	
7	S1_90545383	4,786,261	0.2	0.4	
8	S1_92199236	6,440,114	0.1	0.12	
9	S1_98916246	13,157,124	0.22	0.1	Latitude
10	S1_109332316	5,100,157	0.16	0.14	
11	S1_109332327	5,100,168	0.21	0.25	
12	S1_110307739	6,075,580	0.21	0.18	
13	S1_122235324	4,783,720	0.25	0.13	
14	S1_131785203	786,955	0.3	0.11	
15	S1_166680858	11,596,004	0.14	0.1	
16	S1_174926422	5,114,912	0.2	0.04	
17	S1_190284134	8,569,870	0.35	0.1	
18	S1_190284138	8,569,874	0.16	0.06	
19	S1_238491065	11,486	0.16	0.15	
20	S1_238491098	11,519	0.36	0.15	V1, V10, V12, V13, V16, V18, Longitude
21	S1_241084639	2280	0.38	0.11	V12, V13, V16, V18, Longitude

The significant SNP genotypes correlated with environmental variables for both the likelihood ratio (G) and Wald tests at the Bonferroni-corrected 0.05 alpha levels are shown. The variable numbers are explained in Additional file 1: Table S1

### Gene ontology annotation of genes at divergent loci

We retrieved 52 non-redundant BeeBase Genes IDs for known gene products most proximal to each of the 83 divergent SNPS, as well as gene names and functions, as listed in Table 4. Given the more extensive gene annotation available for *D. melanogaster*, we converted these 52 genes to homologs, resulting in 38 recognized genes examined in the GeneMania tool for functional enrichment. The eleven significantly enriched functional categories are listed in Table 7. Utilizing the same *D. melanogaster* gene list, g:Profiler highlighted three genes participating in one significant Biological Process: chitin-based embryonic cuticle biosynthetic process (GO:0008362, *P* = 3.69e-02).

**Table 7:** Functional enrichment for the genes closest to the divergent SNP loci between *A.m. capensis* and *A.m. scutellata*

GO Functional Category	FDR	# of genes in network	# of genes in genome
Plasma membrane region	0.00722	6	70
Regulation of growth	0.0252	8	215
Embryo development ending in birth or egg hatching	0.0341	7	181
Embryonic development via the syncytial blastoderm	0.0341	7	170
Membrane region	0.0341	6	120
Regulation of synaptic growth at neuromuscular junction	0.0344	5	73
Morphogenesis of an epithelium	0.0360	8	273
Regulation of synapse assembly	0.0360	5	77
Regulation of nervous system development	0.0401	7	204
Regulation of synapse organization	0.0541	5	89
Apicolateral plasma membrane	0.0613	3	17

### Associations between genetic and environmental parameters

Analysis of allele frequency variation of 47 divergent SNPs within *A.m. capensis* (337 individuals and 15,564 non-missing genotypes) generated a total of 2961 models, within which 25 SNPs were significantly correlated (Bonferroni-corrected *P* < 0.05) with one or more environmental parameters (191 significant associations, 6.5% of the total) (Table 5). Of those, 16 loci were significantly related to the longitude of the apiary where the sample was collected; one was significantly related to latitude of the same. Six of 25 loci were significantly associated with isothermality and one with annual mean temperature. Furthermore, six of the 25 loci were exclusively correlated with temperature, and six with precipitation. The strong locus-environment associations were observed in eight SNP loci including S1_108684700, S1_108684756, S1_109332327, S1_110951124, S1_110951168, S1_110951197, S1_111171339, S1_111171425 and S1_111285658 (Table 5).

Within *Apis mellifera scutellata* (73 individuals and 21 divergent loci) generated 1533 non-missing genotypes with a total of 1323 models, of which four SNPs were significantly correlated (Bonferroni-corrected *P* < 0.05) with one or more environmental parameters (21 significant associations, 1.6% of the total) (Table 6). Two loci were significantly related to longitude and two with latitude. The strongest locus-environment association was observed in two SNPs: S1_238491098 and S1_241084639. The highest contribution by percentage among the environmental parameters was to temperature and precipitation in the warmest seasons.

### Model-based Bayesian population structure and genetic differentiation between *A.m. capensis* and *A.m. scutellata*

**Fig. 5:**
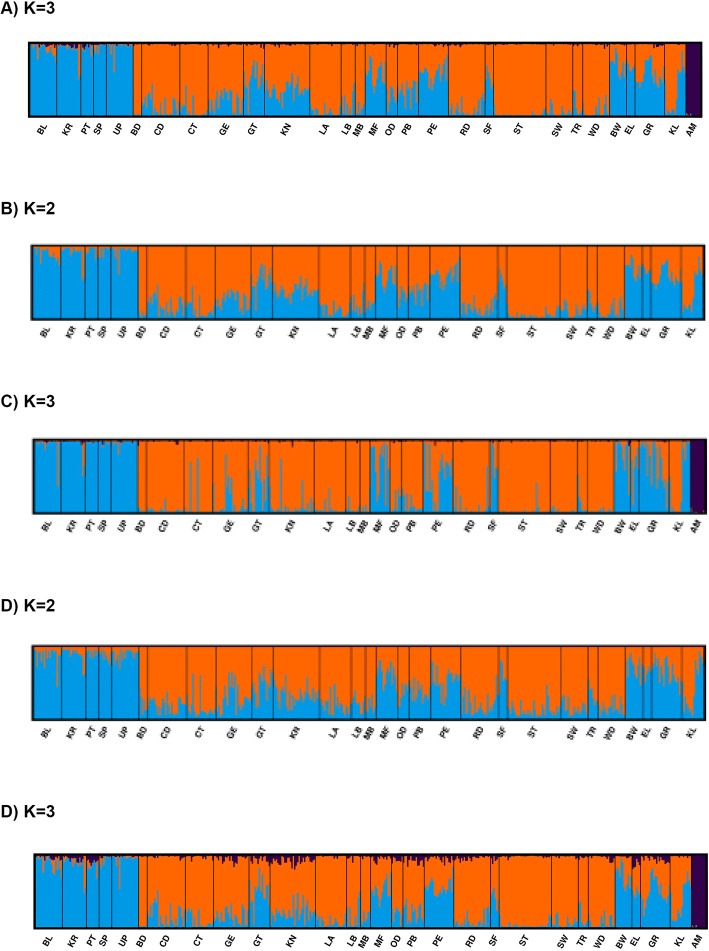
A model-based Bayesian population structure of 474 honey bees from the Republic of South Africa using admixture pattern as included in STRUCTURE program. The Y-axis in each plot indicates the estimated membership coefficients for each individual. Each individual’s genotype is represented by a single vertical line, which is partitioned into colored segments corresponding to the estimated membership in the two or three groups. AM in each plot represents European *Apis mellifera*. **a** Population clustering based on 2449 SNP loci with European AM. **b** Population clustering based on 2449 SNP loci without European AM. **c** Population clustering based on 83 divergent SNP loci with European AM. **d** Population clustering based on 83 divergent SNP loci without European AM

**Fig. 6:**
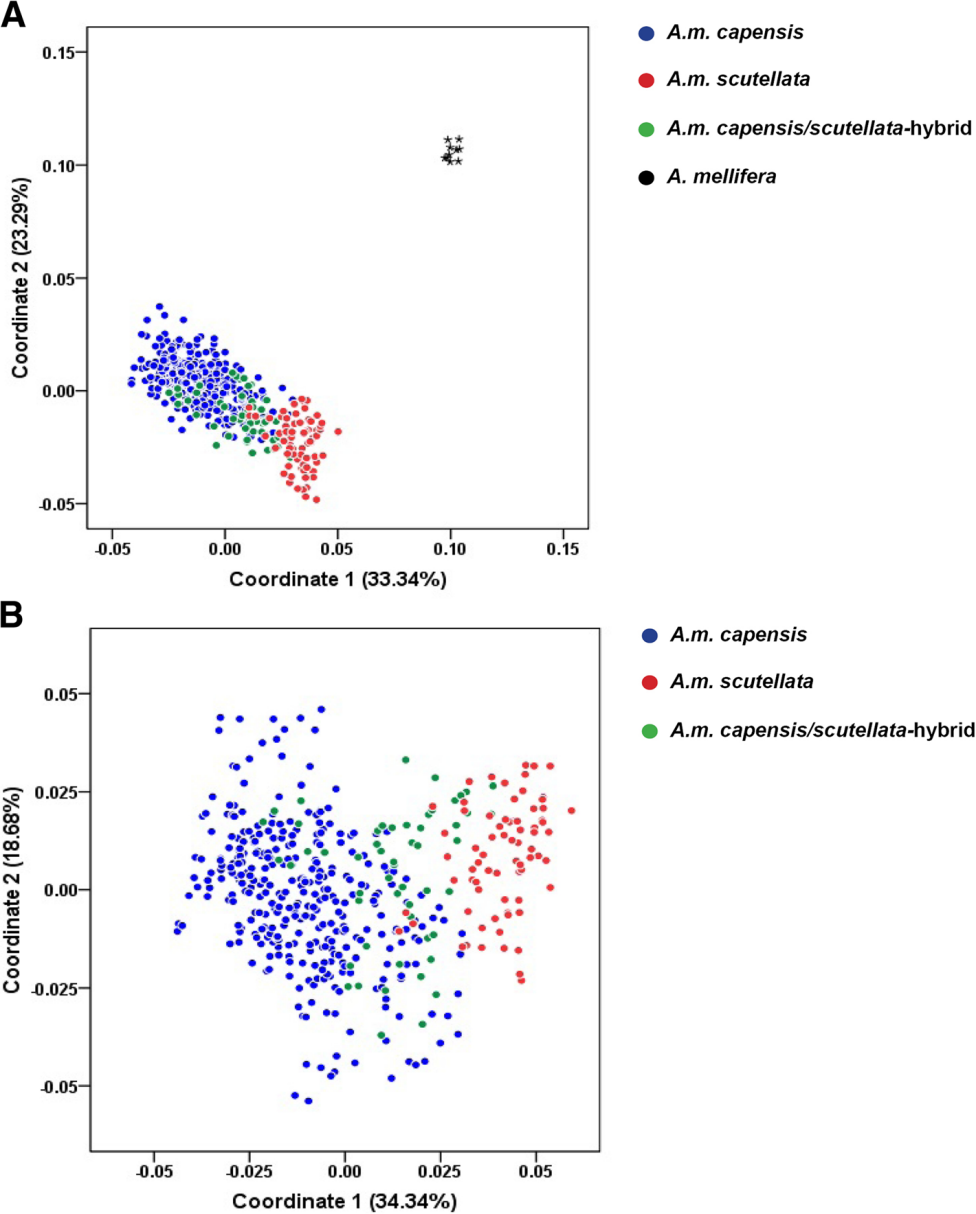
Clustering pattern obtained by discriminant analysis of principal coordinates using 2449 SNP loci with 474 honey bees collected from 28 geographical regions in the Republic of South Africa. Each group is shown by different colors and the colored circle dots in the figures represent individuals of each subspecies. European *Apis mellifera* are indicated with a star. **a** Clustering plot of all individuals with European *A. mellifera* and **b** without European *A. mellifera*

The model-based Bayesian population structure was determined for the two sets of data, all 2449 SNPs, and the 83 divergent SNPs. Using 2449 SNP loci, the delta *K* method suggested three genetic groups (*K* = 3) with inclusion of European subspecies of *A. mellifera* (Fig. 1, Additional file 8: Table S3). When we excluded European *A. mellifera* (*n* = 10), two genetic groups were observed according to the delta *K* calculation as determined by the Harvester method (*K* = 2) (Fig. 5). *Apis mellifera scutellata* constituted a distinct genetic group with genetic homogeneity across the regions (Fig. 5). In *A.m. capensis*, nine regions (CD, CT, GE, KN, LA, PE, RD, ST and WD) confirmed the occurrence of distinct genetic groups, and variable degrees of genetic heterogeneity were observed across its natural distribution in the Cape region (Fig. 5). Hybrid regions showed a mixture pattern of genotypes, with a variable percent of *K* membership in each region (Fig. 5). When we examined individual membership across K populations, *A.m. scutellate* possessed, on average, 5% of K1, 93% of K2, and 2% of K3 ancestry. In contrast, *A.m. capensis* was assigned primarily to K1 (78%), with lesser contributions from K2 (21%) and K3 (K3). The hybrid population appropriately contained roughly equivalent proportions of the two primary Ks: 44% K1, 55% K2 and 1% K3. The PCoA analysis confirmed these results, positioning *A.m. scutellata* and *A.m. capensis* into two clusters with hybrids placed at intermediate positions (Fig. 6). European *A. mellifera* clustered into a single distant group. The first and second component accounted for 23.29% and 33.34% of the variance, respectively. With European *A. mellifera* excluded, 20% of the individual *A.m. scutellata* and hybrids overlapped in the PCoA plot, 44.5% of *A.m. capensis* and hybrids overlapped, 4% of *A.m. capensis* and *A.m. scutellata* overlapped, and 4% overlapped between all three groups.

**Fig. 7:**
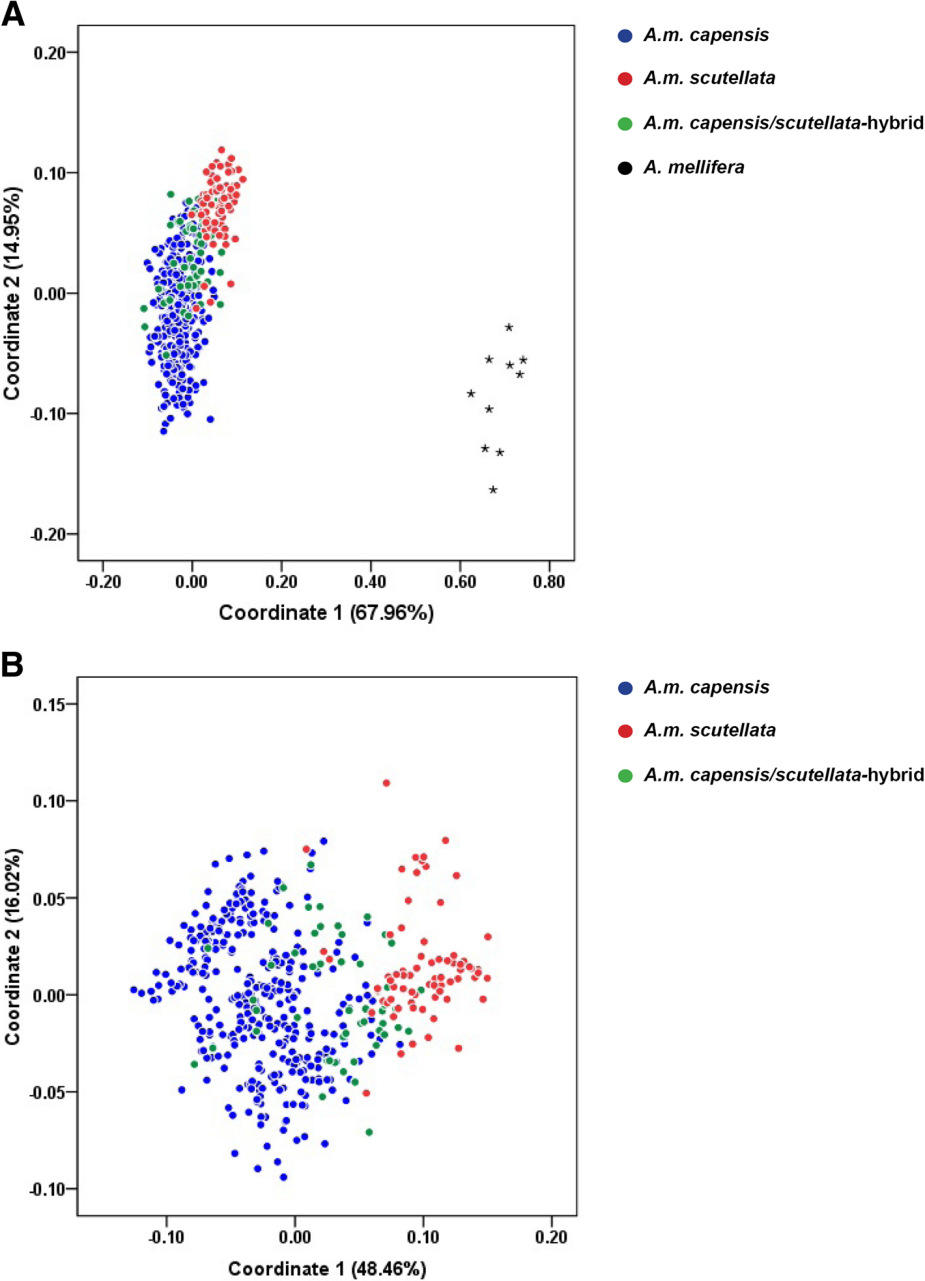
Clustering pattern obtained by discriminant analysis of principal coordinate using 83 divergent SNP loci with collected from 28 geographical regions in the Republic of South Africa. Each group is shown by different colors and the colored circle dots in the figures represent individuals of each subspecies. European *Apis mellifera* are indicated with a star. **a** Clustering plot of all individuals with European *A. mellifera* and **b** without European *A. mellifera*

Using the reduced set of just 83 divergent SNP loci and including European *A. mellifera* in the analysis, *A.m. scutellata* and *A.m. capensis* revealed a similar delta *K* value consistent with the pattern produced with the 2449 SNP set, supporting three distinctive genetic clusters (*K* = 3) across individuals (Fig. 5). Two genetic groups were defined (*K* = 2), when European *A. mellifera* were excluded. In contrast, the PCoA analysis displayed increased resolution with the reduced marker set, capturing 14.95% and 67.96% of the variance in the first and second components respectively (Fig. 7). With European *A. mellifera* excluded, 37% of the individual *A.m. scutellata* and hybrids overlapped in the PCoA plot, 69% of the *A.m. capensis* and hybrids overlapped, 0.2% of the *A.m. capensis* and *A.m. scutellata* overlapped, and 9% of all three groups overlapped.

Using the set of 2449 SNP loci, a low level of population differentiation (*F*_ST_) was observed within each group, estimated at 0.035 for *A.m. capensis* and 0.04 for *A.m. scutellata*.

We observed the highest significant pairwise genetic differentiation between BD and all other regions (0.07 to 0.12, *P* < 0.05). The lowest *F*_ST_ values observed among other pairs of regions ranged from 0.02 to 0.09, *P* < 0.05 (Table 8). *Apis mellifera capensis* and *A.m. scutellata* revealed a lower value of genetic differentiation using all 2449 SNP loci, although they are distinctly clustered in the structure plots. AMOVA results revealed that most of the total genetic variation occurred within populations (93%, *P* < 0.001), while only 3% was attributed across populations (*P* < 0.001).

**Table 8:** Pairwise *F*_ST_ values computed using 2449 SNPs from honey bees collected from 28 geographical regions in the Republic of South Africa. European *A. mellifera* (AM) was included as a single region in the dataset

	BL	KR	PT	SP	UP	OD	PB	PE	RD	SF	ST	SW	TR	WD	MF	MB	LB	LA	KN	GT	GE	CT	CD	BD	GR	BW	EL	KL	AM
BL	0																												
KR	0.03	0.00																											
PT	0.04	0.04	0.00																										
SP	0.04	0.05	0.06	0.00																									
UP	0.03	0.04	0.05	0.03	0.00																								
OD	0.05	0.05	0.06	0.06	0.05	0.00																							
PB	0.05	0.05	0.06	0.06	0.05	0.03	0.00																						
PE	0.04	0.04	0.05	0.06	0.04	0.03	0.04	0.00																					
RD	0.05	0.05	0.06	0.06	0.05	0.03	0.04	0.04	0.00																				
SF	0.04	0.04	0.05	0.05	0.04	0.04	0.03	0.03	0.03	0.00																			
ST	0.05	0.05	0.06	0.07	0.06	0.03	0.04	0.04	0.03	0.04	0.00																		
SW	0.05	0.06	0.06	0.07	0.06	0.03	0.04	0.04	0.03	0.04	0.03	0.00																	
TR	0.07	0.07	0.09	0.08	0.07	0.05	0.06	0.06	0.06	0.06	0.05	0.05	0.00																
WD	0.06	0.06	0.07	0.07	0.06	0.03	0.04	0.04	0.03	0.04	0.03	0.03	0.06	0.00															
MF	0.04	0.04	0.05	0.06	0.04	0.04	0.04	0.04	0.04	0.03	0.04	0.04	0.06	0.05	0.00														
MB	0.05	0.05	0.07	0.06	0.05	0.04	0.04	0.04	0.03	0.04	0.03	0.04	0.06	0.03	0.04	0.00													
LB	0.05	0.05	0.06	0.06	0.05	0.03	0.03	0.04	0.03	0.04	0.02	0.03	0.06	0.03	0.04	0.03	0.00												
LA	0.05	0.05	0.06	0.06	0.05	0.03	0.03	0.04	0.03	0.03	0.03	0.03	0.05	0.03	0.04	0.03	0.03	0.00											
KN	0.04	0.05	0.05	0.06	0.05	0.03	0.03	0.04	0.03	0.03	0.03	0.03	0.06	0.03	0.03	0.04	0.03	0.03	0.00										
GT	0.04	0.04	0.05	0.06	0.04	0.03	0.04	0.03	0.03	0.03	0.04	0.04	0.06	0.04	0.03	0.03	0.03	0.03	0.04	0.00									
GE	0.04	0.04	0.05	0.06	0.04	0.03	0.03	0.04	0.02	0.03	0.03	0.03	0.05	0.03	0.03	0.03	0.03	0.03	0.02	0.03	0.00								
CT	0.05	0.05	0.06	0.06	0.05	0.03	0.03	0.04	0.03	0.03	0.02	0.03	0.05	0.03	0.04	0.03	0.03	0.02	0.03	0.03	0.02	0.00							
CD	0.04	0.05	0.06	0.05	0.05	0.03	0.04	0.04	0.03	0.03	0.03	0.03	0.05	0.03	0.04	0.03	0.03	0.02	0.03	0.03	0.03	0.02	0.00						
BD	0.10	0.10	0.12	0.12	0.10	0.08	0.08	0.08	0.07	0.09	0.07	0.07	0.11	0.07	0.08	0.08	0.08	0.07	0.08	0.08	0.07	0.08	0.07	0.00					
GR	0.03	0.04	0.05	0.05	0.04	0.03	0.04	0.03	0.03	0.03	0.04	0.04	0.06	0.04	0.03	0.04	0.03	0.03	0.03	0.03	0.03	0.03	0.03	0.08	0.00				
BW	0.04	0.05	0.05	0.05	0.04	0.05	0.05	0.04	0.05	0.04	0.05	0.05	0.07	0.05	0.04	0.05	0.05	0.04	0.05	0.04	0.04	0.04	0.04	0.09	0.04	0.00			
EL	0.04	0.04	0.05	0.06	0.04	0.03	0.04	0.04	0.04	0.03	0.04	0.04	0.07	0.04	0.03	0.04	0.04	0.04	0.03	0.03	0.03	0.04	0.04	0.09	0.03	0.04	0.00		
KL	0.05	0.05	0.06	0.06	0.05	0.04	0.05	0.05	0.04	0.04	0.04	0.05	0.06	0.05	0.05	0.04	0.04	0.04	0.04	0.04	0.04	0.04	0.04	0.09	0.04	0.05	0.05	0.00	
AM	0.23	0.22	0.26	0.27	0.23	0.27	0.25	0.23	0.22	0.29	0.23	0.25	0.30	0.24	0.24	0.28	0.26	0.24	0.22	0.24	0.22	0.24	0.22	0.35	0.22	0.25	0.28	0.26	0.00

The geographical abbreviations are shown in Table 1. The significant values of all pairwise comparisons are based on Bonferroni correction (*P* < 0.05)

## Discussion

Morphological studies revealed two distinct morphoclusters of honey bee colonies in RSA [9, 71, 72]. *Apis mellifera capensis* became distinct from other subspecies in Africa due to three phenotypic traits (thelytoky, spermatheca size, and ovariole number) [23]. We found some degree of overlap in the clustering patterns of the two subspecies in RSA, as is observed among other African honey bee subspecies [36]. Our wing geometry, standard morphometric and genomic data supported a clear differentiation between *A.m. capensis* and *A.m. scutellata*, though there were no individual variables that alone predicted this separation. Our study demonstrated that 17 wing geometry and standard morphometric parameters can be used to separate the bees into three clusters coinciding with their subspecies and hybrid distributions. We found that honey bee populations from several regions fell outside of the confidence ellipses and instead positioned at the intermediate regions, as expected for hybrids [4, 9].

With the aid of high-throughput sequencing technologies, it has become possible to genotype a large number of samples economically, this in order to determine genetic diversity, population structure and degree of introgression in different honey bee populations [73, 74]. Here, we utilized GBS technology to infer genetic diversity and population structure in a large collection of honey bees from the Republic of South Africa. To our knowledge, this is the first attempt to use GBS, in comparison with wing geometry and standard morphometric parameters, to characterize the population structure and identify ancestry informative markers that can be applied to distinguish between *A.m. capensis* and *A.m. scutellata* in research and production settings. GBS provided a total of 2449 highly informative SNP markers based on very stringent quality criteria. We found the maximum evolutionary divergence between the African subspecies of honey bees and the European honey bees we tested.

The majority of the SNPs identified in the examined honey bees exhibited a high degree of polymorphism. The average level of polymorphic SNP markers observed in *A.m. capensis*, *A.m. scutellata* and hybrids (*N*_p_ = >80%) was higher than that previously reported for each subspecies of *A. mellifera* (*N*_p_ = 40%) [75]. The average number of SNP polymorphisms across the honey bees we collected in the RSA was higher than those observed in four of the evolutionary groups (A, M, C and O) of honey bees (*N*p = 84.5% vs 30%) [75]. The large SNP variation we detected could be due to number of SNPs tested, the number of honey bees genotyped and geological history of honey bees in Africa.

Genetic diversity based on expected heterozygosity was the highest in almost all regions of *A.m. capensis*, *A.m. scutellata* and hybrids, with an average of *H*_exp_ = 0.23. The highest level of heterozygosity previously reported in the honey bee literature was observed in African bees (*H*_obs_ = 0.12) [73]. This is consistent with previous studies, suggesting that African honey bee subspecies exhibit high genetic diversity, most likely due to their large effective population size, low level of inbreeding between lineages, and lack of population bottlenecks incurred during quaternary ice ages [75–79]. The SNP heterozygosity values reported across regions in our study were lower than those obtained using microsatellite markers [8, 29]. These differences between the two techniques could reflect the multi-allelic nature of microsatellite markers [80]. In one study conducted by Fuller et al. [79], the heterozygosity level observed in the Forkhead Box Protein O (*Foxo*, GB48301) gene was greater in savannah honey bees (likely *A.m. scutellata*) than in desert honey bee populations (likely *A.m. yemenitica* and *A.m. simensis*) in Kenya.

The clustering pattern from the STRUCTURE analysis of 2449 SNPs illustrated shared ancestry correlating to the two known subspecies in the RSA, and was mirrored by the PCoA. These methods distinguished between *A.m. capensis* and *A.m. scutellata*, supporting the idea of two subspecies with distinctive physiological and behavioral differences [9, 79]. In contrast, a set of ancestry informative markers distinguishing these two subspecies could not be found in several recent studies examining both genomic and whole mitochondrial genomes [3, 81–83]. Recently, the effects of Africanization on the genome diversity of 32 Africanized honey bees from Brazil was determined [84]. In that study, signals of positive selection on chromosome 11 indicative of adaptive evolution in the Africanized honey bee population were identified. The authors concluded that African Brazilian honey bee populations are indistinguishable from African ancestry because these two populations have not sufficiently diverged.

Our success in genetically identifying the two subspecies is likely due to the sizable number of individuals we analyzed, and the use of SNP markers specifically derived from the target populations [3, 81, 82]. Our findings are consistent with a study conducted on 11 honey bees collected from four distinct ecological regions (savannah, coast, desert and mountain) in Kenya. Those authors concluded that *A.m. scutellata* in savannah regions can be distinguished from other honey bees based on the phylogenetic analysis of complete mitochondrial genome sequences [79].

The performance of outlier approaches to differentiate honey bee populations has been investigated by others [74, 82]. Here, the outlier analysis suggested that 83 SNPs were potential candidates for use to differentiate *A.m. capensis* and *A.m. scutellata*. Our STRUCTURE and PCoA analyses using 83 divergent loci enhanced the resolution power of SNPs used to discriminate the two subspecies. We suggest that the accuracy and robustness of these markers should be determined on randomly collected samples from RSA. This could validate the discriminatory power of the divergent loci.

The detected signature of admixture within *A.m. scutellata* could be due to the fact that *A.m. scutellata* differs in genetic characteristics from *A.m. capensis*. Unique clustering of *A.m. scutellata* was also observed in phylogenetic analysis based on whole genome data of honey bee populations in Kenya [79]. We found several genomic regions under selective pressure within *A.m. capensis*, allowing reliable assignment of individuals to the population of origin and providing effective tools to identify pure *A.m. capensis* colonies in RSA. We propose that these 83 markers may be utilized effectively for the identification of both subspecies, a critical application for both research and agricultural efforts. These 83 divergent loci may be under natural selection for physiological and behavioral characters adaptive to the native environments of both subspecies.

A functional analysis highlighted processes involved in neurology/behavior and growth/development which were among the most rapidly evolving genes identified in the two subspecies. *Apis mellifera scutellata* and *A.m. capensis* are the two divergent honey bee subspecies that exhibit distinct biological functions in RSA [9, 16–19]. Indeed, these subspecies differ in several aspects of behavior maturation in a presumably adaptive way, including foraging activity and defensive behaviors [9, 12, 15, 17, 27, 28]. The defensive behavior and colony usurpation tendencies of *A.m. scutellata* and thelytoky in *A.m. capensis* are the most heritable traits supporting the functional behavior in this study [12, 15, 17]. Foraging, which encodes a cyclic G-dependent protein kinase, affects feeding and food gathering–related activities in both honey bees and *Drosophila melanogaster* [85, 86].

Honey bees exhibit a suite of diverse behaviors in social environment and several hundred genes have been closely associated with brain function and physiological behaviors in bees [86]. Several genes have been found to regulate neuronal function and behavior, for example the gene metabotropic GABA-B receptor subtype 1 [87]. Chemical signaling is used to coordinate the behavior and physiology of colony members. Changes in the protein-coding sequence are possibly related to the evolution of the chemical communication system found in honey bees [88].

We found several genes of the divergent loci shown to impact embryonic development and growth. Eusocial insects have remarkably diverse exocrine gland functions and produce many novel glandular secretions, including pheromones, brood food, and antimicrobial compounds [89, 90].

Genes involved in caste differentiation, worker development and reproduction in both subspecies are the most prominent examples of gene families gaining diverse functions through the social interactions [91]. Our results provide an avenue for linking specific genetic changes to the functional evolution in these bees. Major challenges in this attempt include determining the genes associated with morphological and behavioral differences between the subspecies and furthering our understanding of how changes in the gene function affect a biological process in these subspecies. However, additional work to measure LD length and haplotype blocks encompassing these divergent loci, and considerable improvement in functional annotation of the reference genome, are needed before conclusive work on selective sweeps can be accomplished in these subspecies.

The distribution and clear differentiation of the two subspecies suggests that they may have been separated by a permanent barrier historically, a barrier likely influenced by environmental conditions such as temperature and precipitation. This is consistent with the literature, which suggests that *A.m. scutellata* prefers warm and dry climates while *A.m. capensis* prefers cooler wetter ones [9, 15]. Indeed, we found significant associations of several divergent SNP loci with environmental parameters, most notably temperature for *A.m. scutellata* SNPs and precipitation for *A.m. capensis* ones. These findings explain the population distribution of the two subspecies along the west-east axis, and supports the occurrence of adaptive divergence related to environmental parameters. Such signals of local adaptation to environmental variables were previously observed in Iberian honey bee populations [92]. We believe that temperature and precipitation could be two important parameters maintaining the population structure of honey bees in RSA. Another factor contributing to genetic divergence of these two species could be isolating differences in the behaviors of *A.m. capensis* and *A.m. scutellata* as demonstrated by Hepburn and Radloff [9], Jaffé et al. [11] and Onions [16].

The low level of differentiation between these two subspecies in our study (*F*_ST_ = 0.06) could be related to a substantial level of gene flow between populations [93]. A second possible reason for low *F*_ST_ values could be attributed to the large population size of *A.m. capensis* and *A.m. scutellata* [9]. The *F*_ST_ values observed in this study for *A.m. capensis* (0.035), *A.m. scutellata* (0.04) and hybrids (0.04), is lower than previously reported for honey bees in Africa [29] and higher than values observed between savannah and desert honey bees in Kenya [79]. We suggest that the lack of any physical barrier between the indigenous ranges of the two subspecies and exchanging of queens and colonies between beekeepers in both areas are contributing factors supporting the admixture pattern within *A.m. capensis* [94, 95]. It was previously noted that honey bee colonies pollinating in hot/dry regions (e.g. *A.m. scutellata*) migrate over large distances to environments with more resources in order to withstand reduced nutrient intake during winter seasons better [79, 84].

We identified 47 and 21 divergent loci for *A.m. capensis* and *A.m. scutellata*, respectively. These results provide evidence for signatures of natural selection in RSA honey bee populations. We demonstrated the relevance of environmental heterogeneity in driving locally adaptive genetic variation within these candidate loci. For divergent loci, temperature and precipitation variables were significantly associated with SNP variants within signatures of selection, highlighting the importance of these environmental factors in adaptation to local conditions [9, 15, 79]. The present findings provide some testable hypotheses for additional experimental analyses of the functional role these genes play in ecological adaptation.

## Conclusions

Considerable genetic diversity is retained within indigenous honey bee populations in RSA. Principal coordinate and population structure analyses clearly differentiated *A.m. capensis* and *A.m. scutellata* populations, and quantified ancestry in hybrid bees, as expected based on their behavioral and ecological characteristics. The differentiation pattern describes the genetic distinctiveness of *A.m. scutellata* and *A.m. capensis* populations. The regional admixture observed in *A.m. capensis* populations represents a unique genetic resource, and an unexploited opportunity, that necessitates initiatives for the sustainable conservation of this subspecies. The significant identification of divergent SNP loci by environmental variables suggests adaptive selection occurring within the RSA honey bee subspecies. The wing geometry and standard morphometric analyses supported grouping the bees into two subspecies, which was consistent with genetic structure. We believe the 83 divergent SNPs discovered here enable distinguishing between *A.m. scutellata* and *A.m. capensis* with improved efficiency and accuracy.

## Additional files

Additional file 1: **Table S1.** Bioclimatic variables used in the distribution model analysis for the two honey bee subspecies from the Republic of South Africa. (DOC 127 kb)

Additional file 2: **Figure S1.** Canonical variance analysis factor loadings of wing geometry and standard morphometric measurements onto Canonical Vector 1 (CV1) (A) and Canonical Vector 2 (CV2) (B), based on subspecies classifications, for 464 measured honey bees collected from the Republic of South Africa. CV1 and CV2 factor loadings all generated the varied sign and contributed in positive and negative values. (TIF 8470 kb)

Additional file 3: **Figure S2.** Canonical variance analysis factor loadings of wing geometry and standard morphometric measurements onto Canonical Vector 1 (CV1) (A) and Canonical Vector 2 (CV2) (B), based on regional classifications, for 464 measured honey bees collected from the Republic of South Africa. CV1 and CV2 factor loadings all generated the varied sign and contributed in positive and negative values. (TIF 8470 kb)

Additional file 4: **Figure S3.** The hierarchical clustering structure of 464 honey bees collected from 28 geographical regions in the Republic of South Africa. The colors indicate different subspecies: blue = *Apis mellifera scutellata* (*N* = 73), red = *A.m. capensis* (*N* = 337) and green = hybrids (*N* = 54). (TIF 622 kb)

Additional file 5: **Table S2.** A pair-wise evolutionary divergence matrix based on a corrected *p*-distance nucleotide model among honey bees in 29 geographical regions in the Republic of South Africa and a reference European *Apis mellifera*. The geographical abbreviations are explained in Table 1. (JPG 270 kb)

Additional file 6: **Figure S4 A, B.** Identification of putative divergent SNP loci under directional selection for *Apis mellifera capensis* based on *F*_ST_ outlier approaches. (A) Hierarchical structure model using Arlequin 3.5. *F*_ST_: locus –specific genetic divergence among the populations; Heterozygosity: measure of heterozygosity per locus. The significant loci are shown with red dots (*P* < 0.01). (B) Finite island model (fdist) by LOGISTAN. Loci under positive selection above 99% percentile are shown in the red area. Loci shown in the gray area are neutral loci and those in the yellow area are under balancing selection. (JPG 592 kb)

Additional file 7: **Figure S5 A, B.** Identification of putative divergent SNP loci under directional selection for *Apis mellifera scutellata* based on *F*_ST_ outlier approaches. (A) Hierarchical structure model using Arlequin 3.5. *F*_ST_: locus –specific genetic divergence among the populations; Heterozygosity: measure of heterozygosity per locus. The significant loci are shown with red dots (*P* < 0.01). (B) Finite island model (fdist) by LOGISTAN. Loci under positive selection above 99% percentile are shown in the red area. Loci shown in the gray area are neutral loci and those in the yellow area are under balancing selection. (JPG 272 kb)

Additional file 8: **Table S3.** Estimated posterior probabilities and delta *K* for each *K* partition. (JPG 571 kb)
