# Supplementary material for: Population genomics and morphometric assignment of western honey bees (Apis mellifera L.) in the Republic of South Africa
Source: BMC Genomics. 2018 Aug 15;19:615. doi: 10.1186/s12864-018-4998-x (PMC6094452; doi:10.1186/s12864-018-4998-x)
Supplement: Supplementary file 1 — Table S1. Bioclimatic variables used in the distribution model analysis for the two honey bee subspecies from the Republic of South Africa. (DOC 127 kb) [file 12864_2018_4998_MOESM1_ESM.doc]

**Additional file 1: Table S1**

| **variable no.** | **meaning** |
| --- | --- |
| V1 | annual mean temperature |
| V2 | mean diurnal range (mean of monthly (maximum temperature - minimum temperature)) |
| V3 | isothermality (V2/V7) |
| V4 | temperature seasonality (standard deviation of monthly temperature) |
| V5 | maximum temperature of warmest month |
| V6 | minimum temperature of coldest month |
| V7 | temperature annual range (V5-V6) |
| V8 | mean temperature of wettest quarter |
| V9 | mean temperature of driest quarter |
| V10 | mean temperature of warmest quarter |
| V11 | mean temperature of coldest quarter |
| V12 | annual precipitation |
| V13 | precipitation of wettest month |
| V14 | precipitation of driest month |
| V15 | precipitation seasonality (coefficient of variation) |
| V16 | precipitation of wettest quarter |
| V17 | precipitation of driest quarter |
| V18 | precipitation of warmest quarter |
| V19 | precipitation of coldest quarter |

**Additional file 5: Table S2.**

|  | **BL** | **KR** | **PT** | **SP** | **UP** | **BD** | **CD** | **CT** | **GE** | **GT** | **KN** | **LA** | **LB** | **MB** | **MF** | **OD** | **PB** | **PE** | **RD** | **SF** | **ST** | **SW** | **TR** | **WD** | **BW** | **EL** | **GR** | **KL** | **AM** |
| --- | --- | --- | --- | --- | --- | --- | --- | --- | --- | --- | --- | --- | --- | --- | --- | --- | --- | --- | --- | --- | --- | --- | --- | --- | --- | --- | --- | --- | --- |
| **BL** |  |  |  |  |  |  |  |  |  |  |  |  |  |  |  |  |  |  |  |  |  |  |  |  |  |  |  |  |  |
| **KR** | 0.01 |  |  |  |  |  |  |  |  |  |  |  |  |  |  |  |  |  |  |  |  |  |  |  |  |  |  |  |  |
| **PT** | 0.01 | 0.01 |  |  |  |  |  |  |  |  |  |  |  |  |  |  |  |  |  |  |  |  |  |  |  |  |  |  |  |
| **SP** | 0.01 | 0.01 | 0.02 |  |  |  |  |  |  |  |  |  |  |  |  |  |  |  |  |  |  |  |  |  |  |  |  |  |  |
| **UP** | 0.01 | 0.01 | 0.01 | 0.01 |  |  |  |  |  |  |  |  |  |  |  |  |  |  |  |  |  |  |  |  |  |  |  |  |  |
| **BD** | 0.03 | 0.03 | 0.04 | 0.04 | 0.03 |  |  |  |  |  |  |  |  |  |  |  |  |  |  |  |  |  |  |  |  |  |  |  |  |
| **CD** | 0.01 | 0.02 | 0.02 | 0.02 | 0.01 | 0.02 |  |  |  |  |  |  |  |  |  |  |  |  |  |  |  |  |  |  |  |  |  |  |  |
| **CT** | 0.01 | 0.02 | 0.02 | 0.02 | 0.01 | 0.03 | 0.01 |  |  |  |  |  |  |  |  |  |  |  |  |  |  |  |  |  |  |  |  |  |  |
| **GE** | 0.01 | 0.01 | 0.02 | 0.02 | 0.01 | 0.02 | 0.01 | 0.01 |  |  |  |  |  |  |  |  |  |  |  |  |  |  |  |  |  |  |  |  |  |
| **GT** | 0.01 | 0.01 | 0.01 | 0.02 | 0.01 | 0.03 | 0.01 | 0.01 | 0.01 |  |  |  |  |  |  |  |  |  |  |  |  |  |  |  |  |  |  |  |  |
| **KN** | 0.01 | 0.01 | 0.01 | 0.02 | 0.01 | 0.03 | 0.01 | 0.01 | 0.01 | 0.01 |  |  |  |  |  |  |  |  |  |  |  |  |  |  |  |  |  |  |  |
| **LA** | 0.01 | 0.02 | 0.02 | 0.02 | 0.01 | 0.02 | 0.01 | 0.01 | 0.01 | 0.01 | 0.01 |  |  |  |  |  |  |  |  |  |  |  |  |  |  |  |  |  |  |
| **LB** | 0.02 | 0.02 | 0.02 | 0.02 | 0.01 | 0.03 | 0.01 | 0.01 | 0.01 | 0.01 | 0.01 | 0.01 |  |  |  |  |  |  |  |  |  |  |  |  |  |  |  |  |  |
| **MB** | 0.01 | 0.02 | 0.02 | 0.02 | 0.01 | 0.02 | 0.01 | 0.01 | 0.01 | 0.01 | 0.01 | 0.01 | 0.01 |  |  |  |  |  |  |  |  |  |  |  |  |  |  |  |  |
| **MF** | 0.01 | 0.01 | 0.01 | 0.02 | 0.01 | 0.03 | 0.01 | 0.01 | 0.01 | 0.01 | 0.01 | 0.01 | 0.01 | 0.01 |  |  |  |  |  |  |  |  |  |  |  |  |  |  |  |
| **OD** | 0.01 | 0.01 | 0.02 | 0.02 | 0.01 | 0.02 | 0.01 | 0.01 | 0.01 | 0.01 | 0.01 | 0.01 | 0.01 | 0.01 | 0.01 |  |  |  |  |  |  |  |  |  |  |  |  |  |  |
| **PB** | 0.01 | 0.02 | 0.02 | 0.02 | 0.02 | 0.03 | 0.01 | 0.01 | 0.01 | 0.01 | 0.01 | 0.01 | 0.01 | 0.01 | 0.01 | 0.01 |  |  |  |  |  |  |  |  |  |  |  |  |  |
| **PE** | 0.01 | 0.01 | 0.01 | 0.02 | 0.01 | 0.03 | 0.01 | 0.01 | 0.01 | 0.01 | 0.01 | 0.01 | 0.01 | 0.01 | 0.01 | 0.01 | 0.01 |  |  |  |  |  |  |  |  |  |  |  |  |
| **RD** | 0.01 | 0.02 | 0.02 | 0.02 | 0.02 | 0.02 | 0.01 | 0.01 | 0.01 | 0.01 | 0.01 | 0.01 | 0.01 | 0.01 | 0.01 | 0.01 | 0.01 | 0.01 |  |  |  |  |  |  |  |  |  |  |  |
| **SF** | 0.01 | 0.01 | 0.01 | 0.01 | 0.01 | 0.03 | 0.01 | 0.01 | 0.01 | 0.01 | 0.01 | 0.01 | 0.01 | 0.01 | 0.01 | 0.01 | 0.01 | 0.01 | 0.01 |  |  |  |  |  |  |  |  |  |  |
| **ST** | 0.01 | 0.02 | 0.02 | 0.02 | 0.02 | 0.02 | 0.01 | 0.01 | 0.01 | 0.01 | 0.01 | 0.01 | 0.01 | 0.01 | 0.01 | 0.01 | 0.01 | 0.01 | 0.01 | 0.01 |  |  |  |  |  |  |  |  |  |
| **SW** | 0.02 | 0.02 | 0.02 | 0.02 | 0.02 | 0.02 | 0.01 | 0.01 | 0.01 | 0.01 | 0.01 | 0.01 | 0.01 | 0.01 | 0.01 | 0.01 | 0.01 | 0.01 | 0.01 | 0.01 | 0.01 |  |  |  |  |  |  |  |  |
| **TR** | 0.02 | 0.02 | 0.02 | 0.02 | 0.02 | 0.03 | 0.02 | 0.02 | 0.01 | 0.02 | 0.02 | 0.01 | 0.02 | 0.02 | 0.02 | 0.01 | 0.02 | 0.02 | 0.02 | 0.02 | 0.02 | 0.02 |  |  |  |  |  |  |  |
| **WD** | 0.02 | 0.02 | 0.02 | 0.02 | 0.02 | 0.02 | 0.01 | 0.01 | 0.01 | 0.01 | 0.01 | 0.01 | 0.01 | 0.01 | 0.01 | 0.01 | 0.01 | 0.01 | 0.01 | 0.01 | 0.01 | 0.01 | 0.02 |  |  |  |  |  |  |
| **BW** | 0.01 | 0.01 | 0.01 | 0.02 | 0.01 | 0.03 | 0.01 | 0.01 | 0.01 | 0.01 | 0.01 | 0.01 | 0.01 | 0.01 | 0.01 | 0.01 | 0.01 | 0.01 | 0.01 | 0.01 | 0.01 | 0.01 | 0.02 | 0.02 |  |  |  |  |  |
| **EL** | 0.01 | 0.01 | 0.01 | 0.02 | 0.01 | 0.03 | 0.01 | 0.01 | 0.01 | 0.01 | 0.01 | 0.01 | 0.01 | 0.01 | 0.01 | 0.01 | 0.01 | 0.01 | 0.01 | 0.01 | 0.01 | 0.01 | 0.02 | 0.01 | 0.01 |  |  |  |  |
| **GR** | 0.01 | 0.01 | 0.01 | 0.02 | 0.01 | 0.03 | 0.01 | 0.01 | 0.01 | 0.01 | 0.01 | 0.01 | 0.01 | 0.01 | 0.01 | 0.01 | 0.01 | 0.01 | 0.01 | 0.01 | 0.01 | 0.01 | 0.02 | 0.01 | 0.01 | 0.01 |  |  |  |
| **KL** | 0.01 | 0.02 | 0.02 | 0.02 | 0.01 | 0.03 | 0.01 | 0.01 | 0.01 | 0.01 | 0.01 | 0.01 | 0.01 | 0.01 | 0.01 | 0.01 | 0.01 | 0.01 | 0.01 | 0.01 | 0.01 | 0.01 | 0.02 | 0.01 | 0.01 | 0.01 | 0.01 |  |  |
| **AM** | 0.07 | 0.07 | 0.07 | 0.08 | 0.08 | 0.1 | 0.08 | 0.08 | 0.08 | 0.07 | 0.08 | 0.08 | 0.08 | 0.08 | 0.08 | 0.08 | 0.08 | 0.08 | 0.08 | 0.08 | 0.08 | 0.08 | 0.08 | 0.08 | 0.08 | 0.07 | 0.07 | 0.08 |  |

**Additional file 8: Table S3.**

| K | Posterior probabilities (Pp) | Delta *K* (*DK*) |
| --- | --- | --- |
| 1 | 96982.3 | – |
| 2 | 94758.1 | 3.836457 |
| 3 | 92159.7 | 206.1506 |
| 4 | 91778.8 | 1.059898 |
| 5 | 91225 | 1.842192 |
| 6 | 90833.2 | 0.111506 |
| 7 | 90427 | 0.904350 |
| 8 | 90131.3 | 0.655487 |
| 9 | 89717.6 | 0.136552 |
| 10 | 89349 | – |
