# Supplementary figures and images for: Population genomics and morphometric assignment of western honey bees (Apis mellifera L.) in the Republic of South Africa
Source: BMC Genomics. 2018 Aug 15;19:615. doi: 10.1186/s12864-018-4998-x (PMC6094452; doi:10.1186/s12864-018-4998-x)

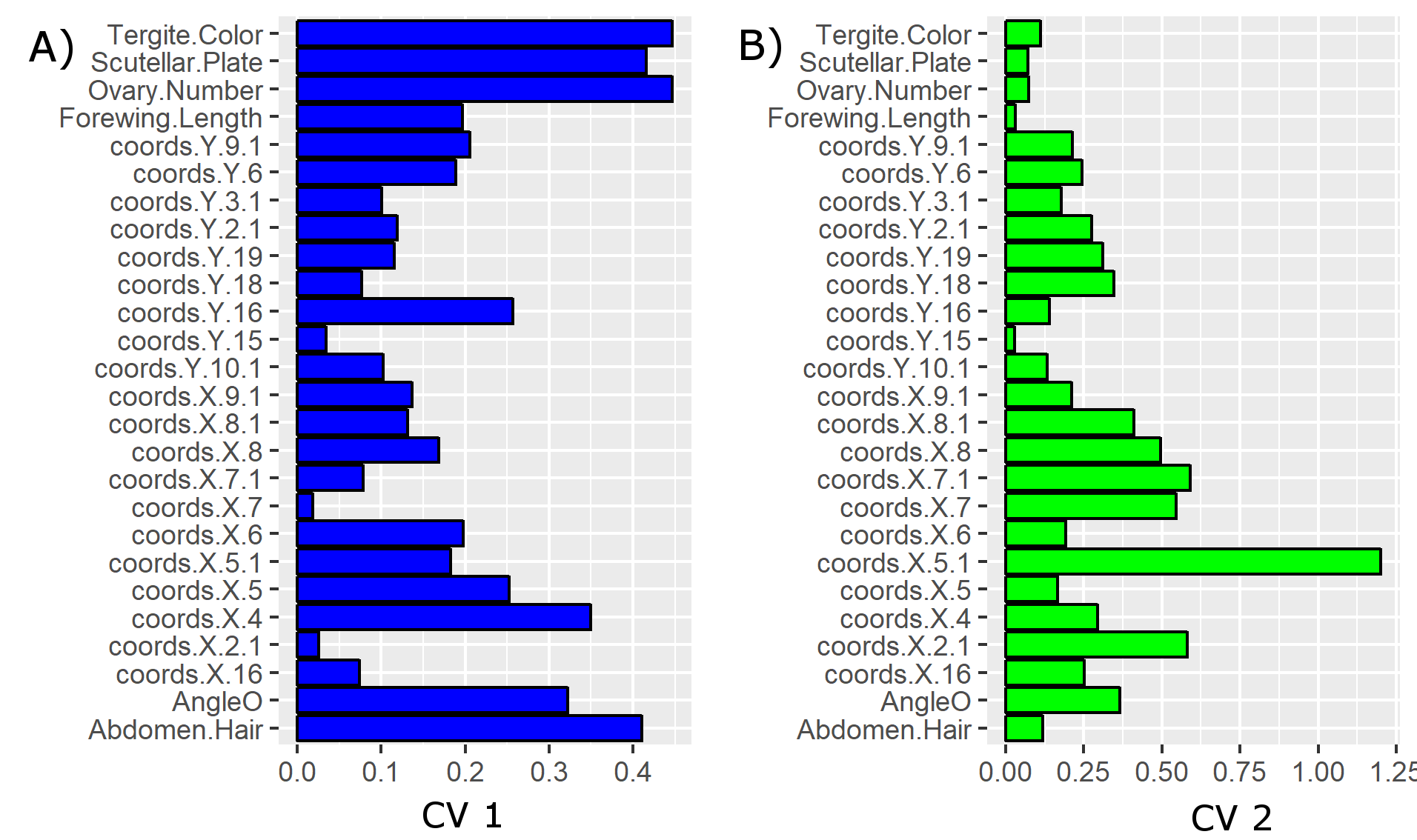

Supplement: Supplementary file 2 — Figure S1. Canonical variance analysis factor loadings of wing geometry and standard morphometric measurements onto Canonical Vector 1 (CV1) (A) and Canonical Vector 2 (CV2) (B), based on subspecies classifications, for 464 measured honey bees collected from the Republic of South Africa. CV1 and CV2 factor loadings all generated the varied sign and contributed in positive and negative values. (TIF 8470 kb) [file 12864_2018_4998_MOESM2_ESM.tif]

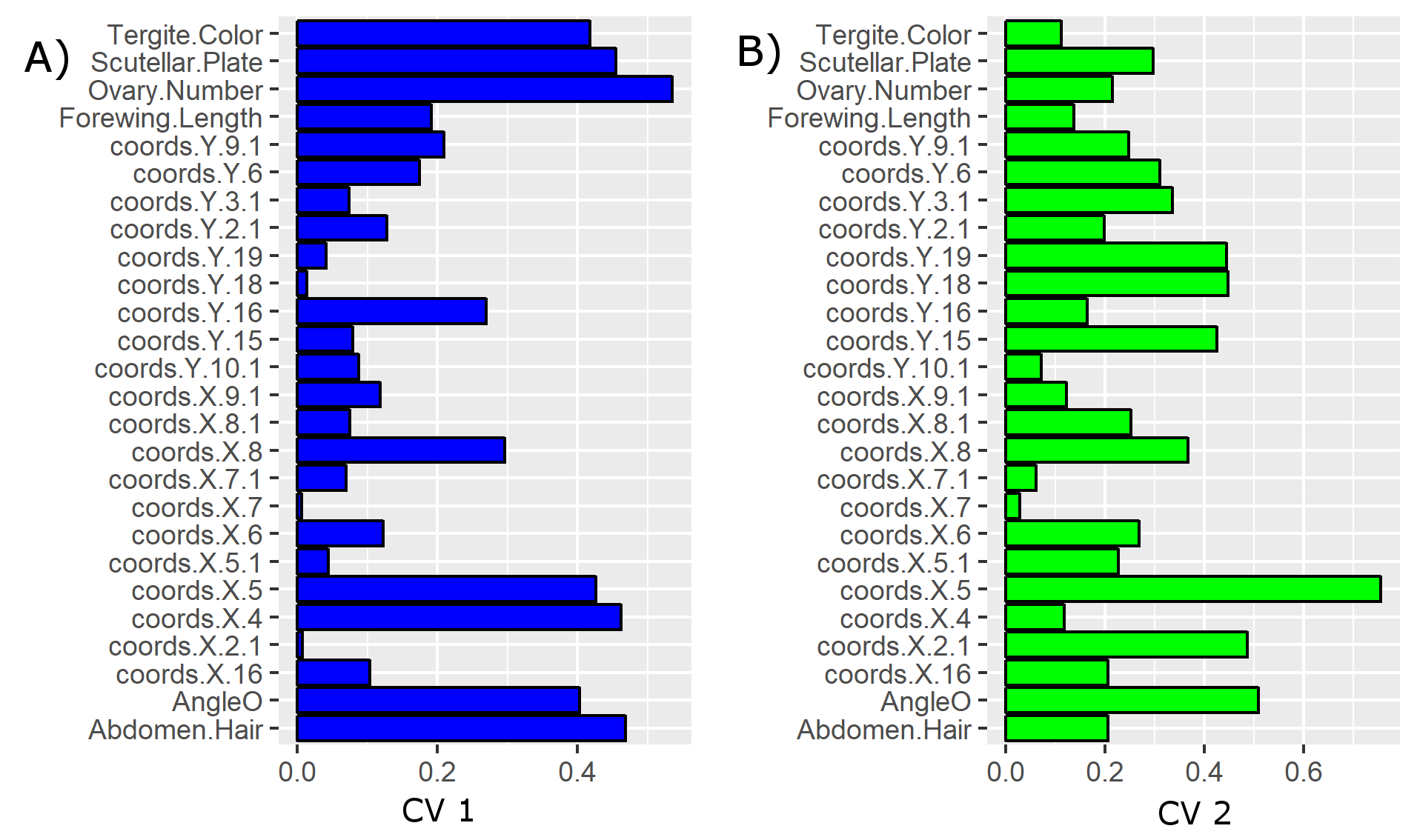

Supplement: Supplementary file 3 — Figure S2. Canonical variance analysis factor loadings of wing geometry and standard morphometric measurements onto Canonical Vector 1 (CV1) (A) and Canonical Vector 2 (CV2) (B), based on regional classifications, for 464 measured honey bees collected from the Republic of South Africa. CV1 and CV2 factor loadings all generated the varied sign and contributed in positive and negative values. (TIF 8470 kb) [file 12864_2018_4998_MOESM3_ESM.tif]

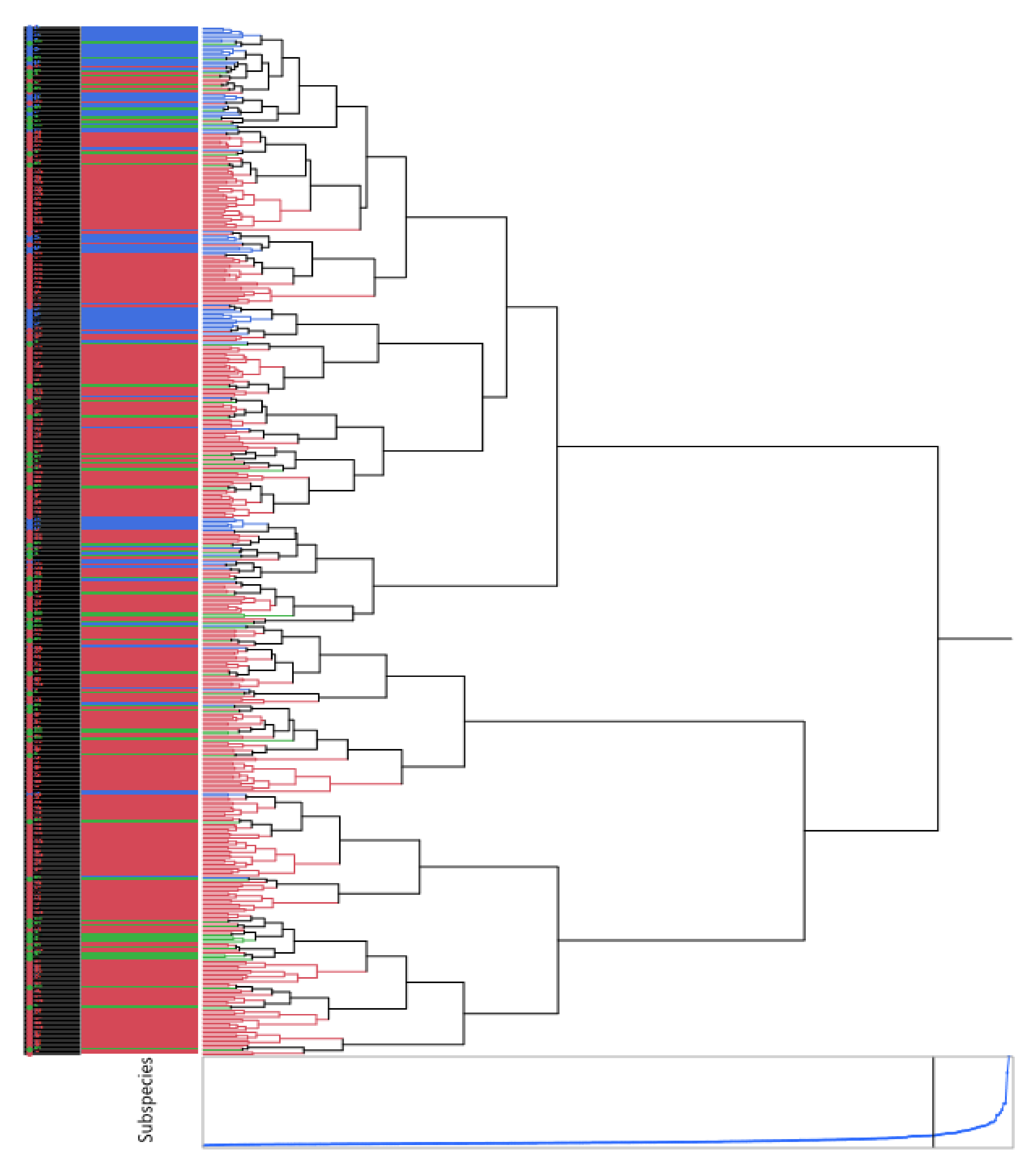

Supplement: Supplementary file 4 — Figure S3. The hierarchical clustering structure of 464 honey bees collected from 28 geographical regions in the Republic of South Africa. The colors indicate different subspecies: blue = Apis mellifera scutellata (N = 73), red = A.m. capensis (N = 337) and green = hybrids (N = 54). (TIF 622 kb) [file 12864_2018_4998_MOESM4_ESM.tif]

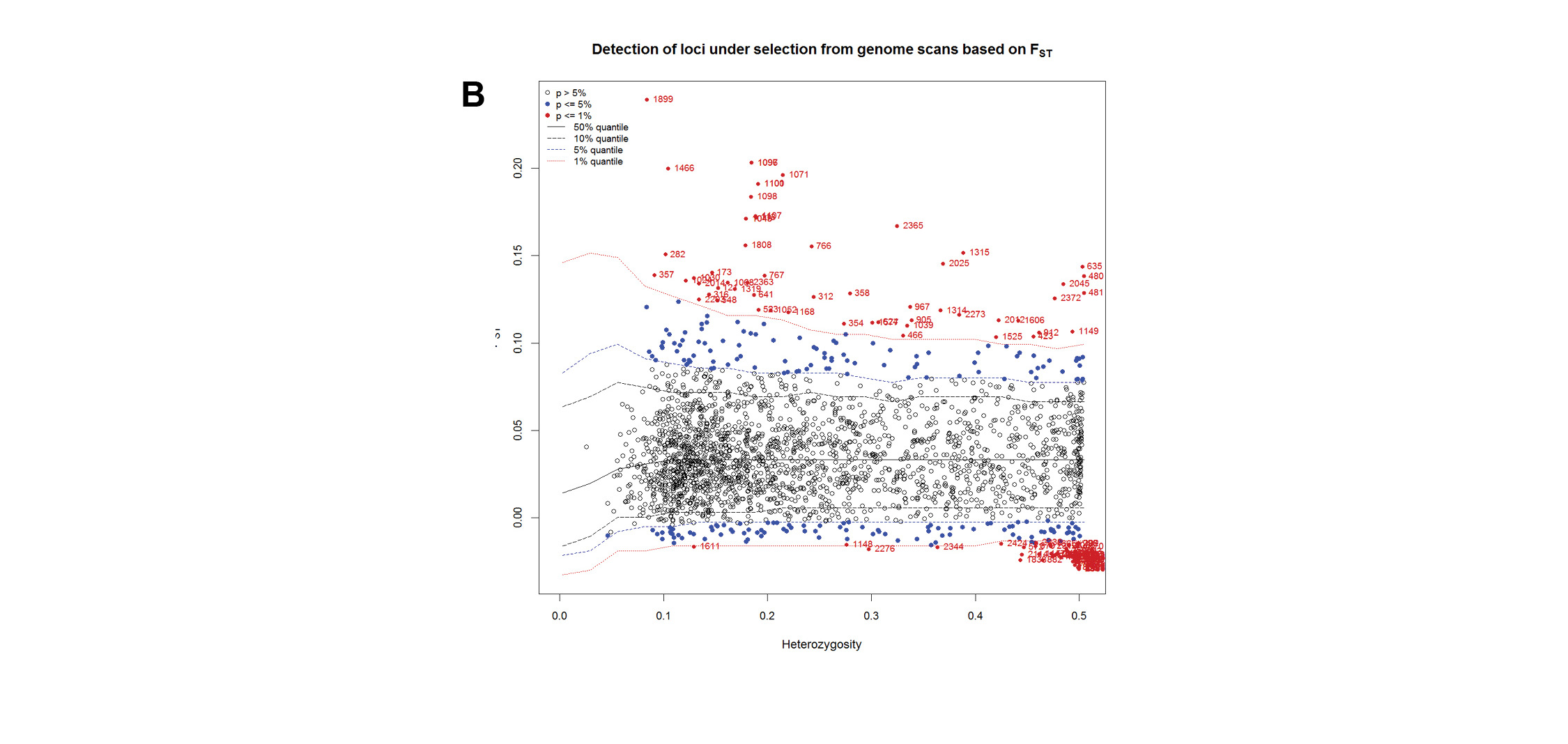

Supplement: Supplementary file 5 — Table S2. A pair-wise evolutionary divergence matrix based on a corrected p-distance nucleotide model among honey bees in 29 geographical regions in the Republic of South Africa and a reference European Apis mellifera. The geographical abbreviations are explained in Table 1. (JPG 270 kb) [file 12864_2018_4998_MOESM5_ESM.jpg]

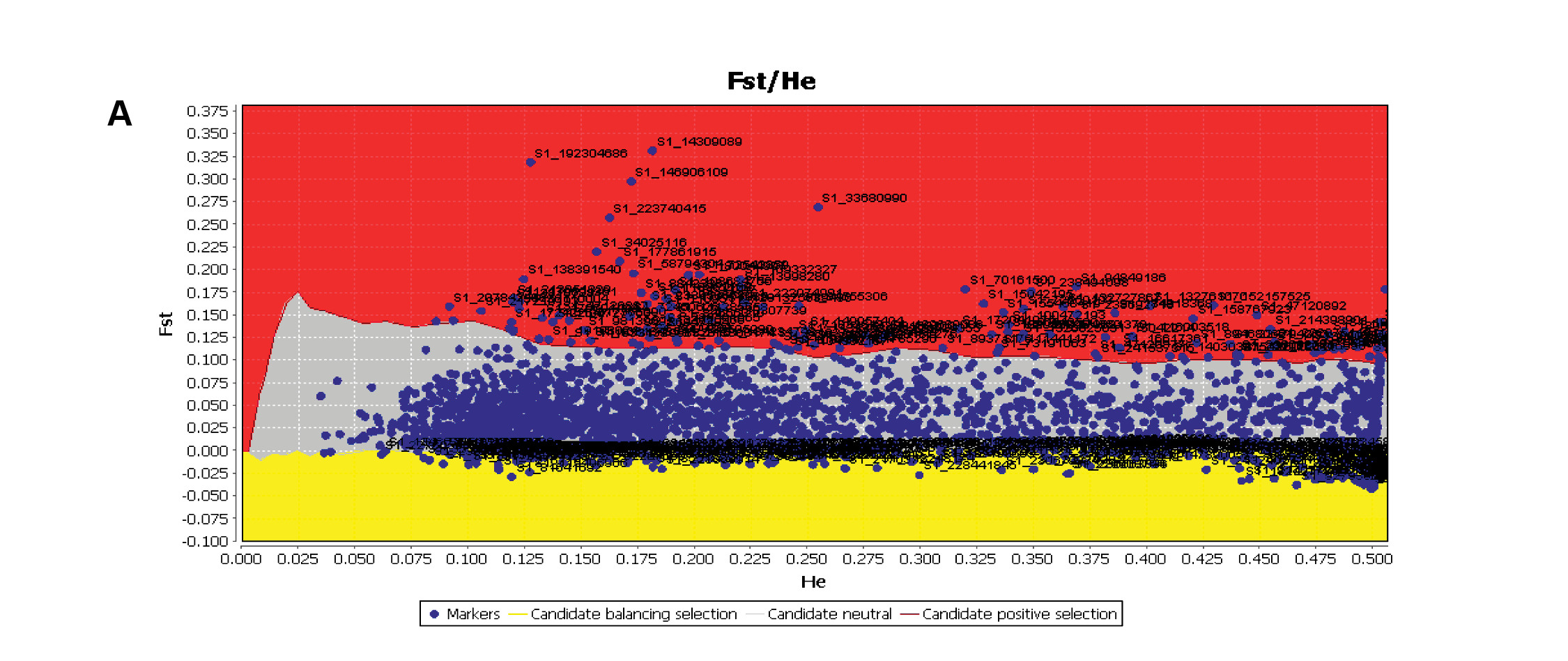

Supplement: Supplementary file 6 — Figure S4 A, B. Identification of putative divergent SNP loci under directional selection for Apis mellifera capensis based on FST outlier approaches. (A) Hierarchical structure model using Arlequin 3.5. FST: locus –specific genetic divergence among the populations; Heterozygosity: measure of heterozygosity per locus. The significant loci are shown with red dots (P < 0.01). (B) Finite island model (fdist) by LOGISTAN. Loci under positive selection above 99% percentile are shown in the red area. Loci shown in the gray area are neutral loci and those in the yellow area are under balancing selection. (JPG 592 kb) [file 12864_2018_4998_MOESM6_ESM.jpg]

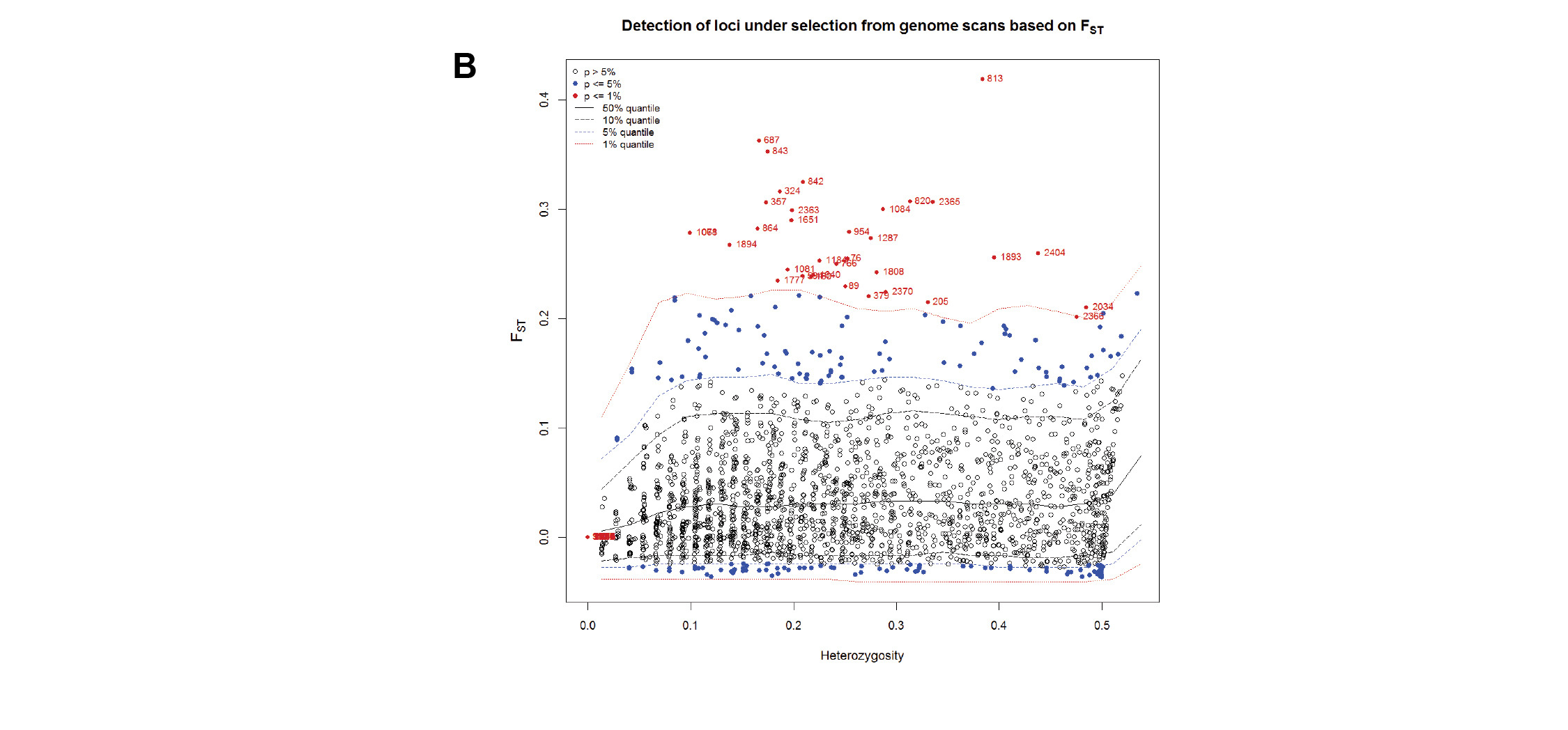

Supplement: Supplementary file 7 — Figure S5 A, B. Identification of putative divergent SNP loci under directional selection for Apis mellifera scutellata based on FST outlier approaches. (A) Hierarchical structure model using Arlequin 3.5. FST: locus –specific genetic divergence among the populations; Heterozygosity: measure of heterozygosity per locus. The significant loci are shown with red dots (P < 0.01). (B) Finite island model (fdist) by LOGISTAN. Loci under positive selection above 99% percentile are shown in the red area. Loci shown in the gray area are neutral loci and those in the yellow area are under balancing selection. (JPG 272 kb) [file 12864_2018_4998_MOESM7_ESM.jpg]

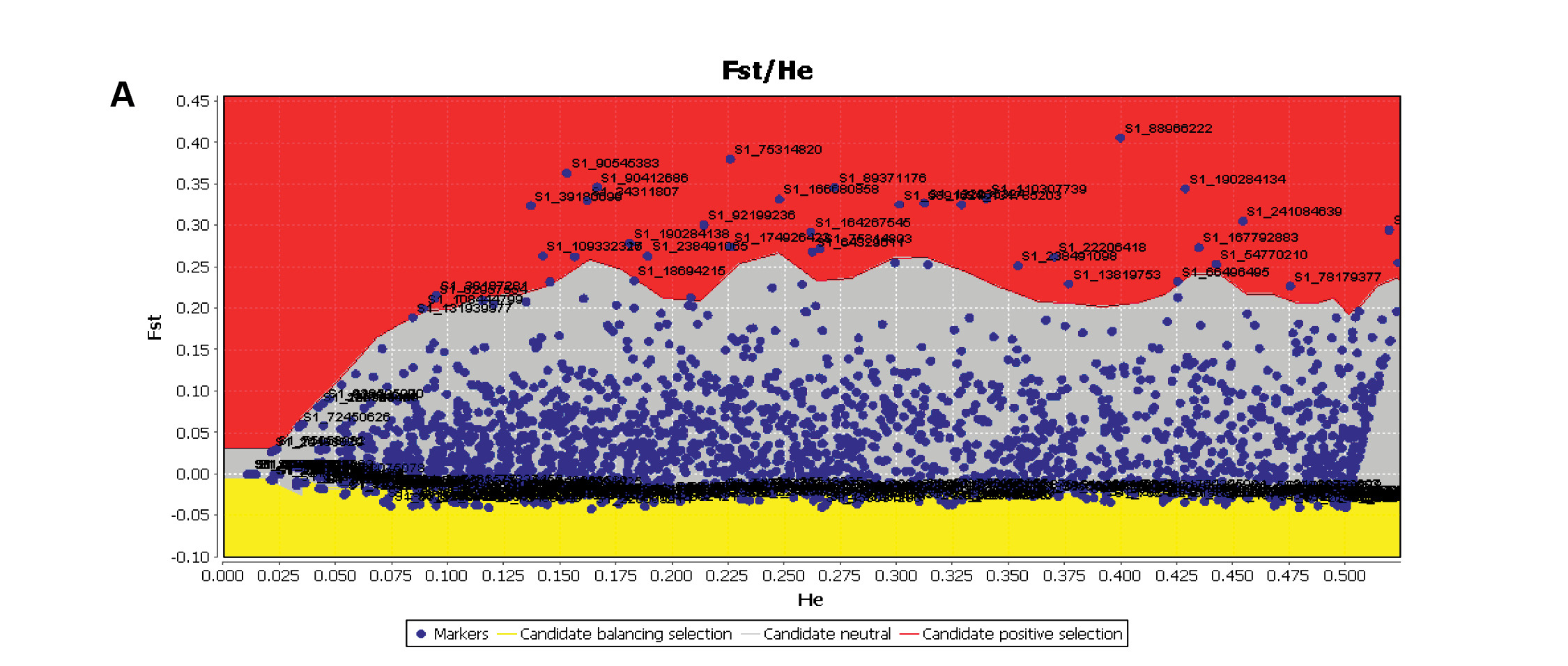

Supplement: Supplementary file 8 — Table S3. Estimated posterior probabilities and delta K for each K partition. (JPG 571 kb) [file 12864_2018_4998_MOESM8_ESM.jpg]
